# Uropathogenic *E. coli* and Hybrid Pathotypes in Mexican Women with Urinary Tract Infections: A Comprehensive Molecular and Phenotypic Overview

**DOI:** 10.3390/cimb46060353

**Published:** 2024-06-13

**Authors:** Manuel G. Ballesteros-Monrreal, Pablo Mendez-Pfeiffer, Bryan Ortíz, Enrique Bolado-Martínez, Maritza Lizeth Álvarez-Ainza, Yessica Enciso-Martínez, Margarita M. P. Arenas-Hernández, Betsaida Diaz-Murrieta, Edwin Barrios-Villa, Dora Valencia

**Affiliations:** 1 Departamento de Ciencias Químico-Biológicas y Agropecuarias, Universidad de Sonora, Caborca CP 83621, Sonora, Mexico; manuel.ballesteros@unison.mx (M.G.B.-M.); pablo.mendez@unison.mx (P.M.-P.); yessica.enciso@unison.mx (Y.E.-M.); betsaida.diaz@unison.mx (B.D.-M.);; 2 Instituto de Investigaciones en Microbiología, Facultad de Ciencias, Universidad Nacional Autónoma de Honduras, Tegucigalpa 11101, Honduras; bryan.ortiz@unah.edu.hn; 3 Departamento de Ciencias Químico-Biológicas, Universidad de Sonora, Hermosillo CP 83000, Sonora, Mexico; enrique.bolado@unison.mx (E.B.-M.); maritza.alvarez@unison.mx (M.L.Á.-A.); 4 Posgrado en Microbiología, Centro de Investigación en Ciencias Microbiológicas, Instituto de Ciencias, Benemérita Universidad Autónoma de Puebla, Ciudad Universitaria, Puebla CP 72570, Pue, Mexico

**Keywords:** urinary tract infection, ERIC, plasmids, ESBL, pathogenicity islands, hybrid pathotypes

## Abstract

Uropathogenic *Escherichia coli* (UPEC) is the main cause of urinary tract infections (UTIs) and carries virulence and resistance factors often found in mobilizable genetic elements, such as plasmids or pathogenicity islands (PAIs). UPEC is part of the extraintestinal pathogenic *E. coli* (ExPEC), but hybrid strains possessing both diarrheagenic *E. coli* (DEC) and ExPEC traits, termed “hypervirulent”, present a significant health threat. This study assessed the prevalence of UPEC PAIs, ExPEC sequence types (ST), DEC genes, carbapenemase and extended-spectrum β-lactamase (ESBL) phenotypes, resistance genotypes, and plasmids in 40 clinical isolates of UPEC. Results showed that 72.5% of isolates had PAIs, mainly PAI IV_536_ (53%). ESBL phenotypes were found in 65% of β-lactam-resistant isolates, with 100% of carbapenem-resistant isolates producing carbapenemase. The predominant ESBL gene was *bla*_CTX-M-2_ (60%), and the most common resistance gene in fluoroquinolone and aminoglycoside-resistant isolates was *aac(6′)Ib* (93%). Plasmids were present in 57% of isolates, and 70% belonged to the ST131 clonal group. Molecular markers for DEC pathotypes were detected in 20 isolates, with 60% classified as hybrid pathotypes. These findings indicate significant pathogenic potential and the presence of hybrid pathotypes in *E. coli* UTI clinical isolates in the Mexican population.

## 1. Introduction

*Escherichia coli* is an important human pathogen that can cause infectious processes of varied severity in different anatomical sites. Its remarkable genomic plasticity has allowed it to adapt to intestinal and extraintestinal environments. According to its genetic content and anatomical site of infection, it has been divided into the following two large groups: the diarrheagenic *E. coli* (DEC) group, which includes at least six different pathotypes, and the extraintestinal pathogenic *E. coli* (EXPEC) group, which includes uropathogenic *E. coli* (UPEC), an etiological agent of urinary tract infections (UTIs) [1,2,3].

UTIs represent one of the most common infectious diseases worldwide and mainly affect women. UTIs can occur both at the bladder level (cystitis) and at the kidney level (pyelonephritis), and, depending on the presence or absence of comorbidities or conditions, such as pregnancy, they can be classified as complicated or uncomplicated [3,4,5]. UTIs represent a critical health problem in Mexico since they are the second cause of morbidity, with more than three million cases per year and a 70% prevalence in women. Urinary tract infections can be caused by a wide variety of pathogens, such as *Proteus mirabilis*, *Klebsiella pneumoniae*, *Enterococci*, *Staphylococcus saprophyticus*, *Pseudomonas aeruginosa*, and even some species of the *Candida* genus. However, the main etiological agent is UPEC [3,6,7]. UPEC possesses a wide variety of virulence factors commonly harbored within large DNA segments called pathogenicity islands (PAIs). PAIs are mobilizable genetic elements (MGEs) that promote the horizontal gene transfer (HGT) of important virulence determinants between isolates. To date, we are aware of the existence of at least thirteen PAIs in the prototype strain of UPEC CFT073 (PAI I–XIII_CFT073_), seven in UPEC 536 (PAI I–VII_536_), and two in UPEC J96 (PAI I–II_J96_) [8,9,10,11,12,13,14,15]. The genetic charge of these PAIs has been described, showing genes associated with adherence, such as the pyelonephritis-associated pilus operon (*papA-K*), F1C pilus (*sfa*), toxins such as alpha-hemolysin (*hlyA* and its gene operon), the secreted autotransporter toxin (*sat*), vacuolating autotransporter toxin (*vat*), cytotoxic necrotizing factor 1 (*cnf-1*), and uropathogenic specific protein (*usp*), genes associated with biofilm and immunoevasion, such as antigen 43 (*agn43*) and capsule (*kpsM*), as well as genes related to iron uptake, such as salmochelin (*iroN*), yersiniabactin (*fyuA*), enterobactin (*iha*), and aerobactin (*iucD*) [16]. These virulence characteristics have allowed UPEC not only to adapt to the environment provided by the urinary tract but also to favor the success of its uropathogenic mechanism.

Another essential feature is the multidrug resistance present in most clinical isolates of *E. coli* recovered from UTI cases. In Mexico, more than 70% of reported clinical isolates of UPEC are classified as multidrug-resistant (MDR) and show resistance to antibiotics used to treat UTIs. β-lactam antibiotics, aminoglycosides, and fluoroquinolones are among the most implemented antibiotics for primary treatment [6,17,18]. Resistance to these drugs can be mediated by enzymes, such as extended-spectrum β-lactamases (ESBL) and acetylases, or by different mechanisms, such as expulsion pumps, and, in the case of fluoroquinolones, proteins that protect the target site [19,20]. According to the reports in Mexico, there is a high prevalence of isolates with ESBL producers’ genotypes and phenotypes [6,21,22,23]. These characteristics and virulence factors can be encoded in MGEs, mainly plasmids, which favor their spread between strains [24,25,26].

On the other hand, the existence of heteropathogenic *E. coli* strains and hybrid pathotypes has been documented; these are important because clinical isolates can possess virulence characteristics of more than one *E. coli* pathotype, which has led to them being consided as hypervirulent strains and can cause more severe infectious processes [27,28].

Despite its importance, information focused on the virulence or resistance characteristics of UPEC and the prevalence of hybrid pathotypes causing UTIs in Mexico is scarce. Therefore, in this work, we detected the presence of MGEs involved with virulence (PAI) in UPEC, the presence of plasmid possibly associated with their antimicrobial resistance, as well as ESBL and carbapenemase production phenotypes in 40 clinical isolates of *E. coli*, previously identified as the etiological agent of UTIs in Mexican population. The presence of molecular markers associated with DEC pathotypes, sequence types (ST), and their adherence patterns was also determined. Finally, phylogenetic analysis was also performed by detection of ERIC (*Enterobacteriaceae* Repetitive Intergenic Consensus) profiles.

## 2. Materials and Methods

### 2.1. Urine Samples and Clinical Isolates

Ninety-eight urine samples were collected from adult women in Sonora, Mexico, from August 2019 to April 2020. Samples were collected in sterile containers by midstream micturition before aseptic indications and kept at 4 °C until they were processed in a time not exceeding 3–4 h. Only one urine sample was collected per patient. The samples were inoculated with nutrient agar to count colony forming unit/mL (CFU/mL) and McConkey agar to identify their colonial morphology. Biochemical identification was carried out by indole, methyl red, Vogues-Prosakuer, and Simmons citrate (IMViC) tests. It included motility, lysine deamination-decarboxylation, urease production, ornithine decarboxylation, and sugar fermentation (glucose, lactose, and sucrose). Additionally, microorganisms biochemically identified as *E. coli* were molecularly confirmed by PCR, amplifying the *ybbW* gene encoding for a particular allantoin receptor of *E. coli* [29,30].

Regarding the inclusion criteria, urine samples with counts below 100,000 CFU/mL from patients exhibiting UTI symptoms or pregnant women were considered. Polymicrobial cultures were also included based on patient characteristics or clinical symptoms of UTIs. However, isolates other than *E. coli* were reported but not included in the study. Additionally, urine samples stored in inadequate containers or for more than 2 h were excluded.

Only 37 urine samples were included in this study according to our inclusion criteria, but in three samples, two different strains of *E. coli* were isolated, which provided us with a total of 40 clinical isolates. The average age of the patients was 47 years, ranging from 19 to 80 years. The most frequent comorbidities were diabetes (28%) and hypertension (25%). Only three patients were pregnant women. As for symptomatology, only 62% presented any symptoms associated with voiding syndrome. It is important to note that a high percentage (70%) of isolates came from women with recurrent UTIs. More detailed information about the clinical characteristics of patients, results for urinalysis, urine cultures, and biochemical or molecular identification of analyzed isolates was previously reported [16].

### 2.2. Genomic DNA Extraction

A single colony of each isolate was inoculated in 3 mL of Müller-Hinton broth (MHB) and incubated for 24 h at 37 °C with constant shaking (150 rpm). Genomic DNA extraction was performed by the alkaline lysis method, according to previously established protocols [31] with some modifications. Briefly, 500 µL of the pre-culture was centrifuged at 16,200× *g* for 10 min; subsequently, 100 µL of a lysozyme (from Sigma-Aldrich (St. Louis, MO, USA); L6876) solution at 20 mg/mL was added, the mixture was vortexed for one minute and then 5 µL of RNAse A (Sigma-Aldrich; R5500) was added, and the mixture was then incubated for 1 h at 37 °C. Subsequently, 50 µL of 20× SDS (Sodium Dodecyl Sulfate from Sigma-Aldrich; L3771-500G) was added and homogenized for 1 min, 200 µL of 5 M NaCl was added and vortexed for 30 s, and the extracts were kept on ice for 5 min and subsequently centrifuged at 16,200× *g* for 20 min and 4 °C. The supernatant was transferred to a new sterile tube, and 700 µL of cold 2-propanol (from Sigma-Aldrich; I9516) was added. The tube was manually shaken for 5 min and centrifuged again for 15 min at 16,200× *g* and 4 °C; the supernatant was discarded, and the DNA pellet was allowed to dry at room temperature for 1 h and then resuspended in 200 µL of nuclease-free water. The extraction product was resolved by electrophoresis in an 0.7% agarose gel, stained with ethidium bromide solution (10 mg/mL), and visualized with a UVP photodocumenter (AnalytikJena, Upland, CA, USA) to determine the integrity of the DNA.

### 2.3. Pathogenicity Islands (PAIs) Detection

The presence of eight pathogenicity islands was detected by polymerase chain reaction (PCR) using the previously established oligonucleotides and conditions reported by Sabate (Table S1) [8]. PCR products were resolved on 2% agarose gels and stained with GelStar (Lonza, Morrinstown, NJ, USA). Each PCR reaction was performed using a GoTaq^®^ Green Master Mix (PROMEGA, Madison, WI, USA) following the manufacturer guidelines.

### 2.4. Plasmid DNA Extraction

Plasmid DNA extraction was performed using previously reported methods [31]. The extraction product was resolved on 2% agarose gel electrophoresis, stained with ethidium bromide solution (10 mg/mL), and visualized with a UVP photodocumenter (AnalytikJena, Upland, CA, USA). The band profiles were analyzed with gel analyzer software 19.1 (www.gelanalyzer.com; accessed on 1 June 2023) using a 1 kb molecular weight marker to normalize the bands and obtain their sizes. For larger plasmids, we used as a control the *E. coli* strain EDL933, which has a 92 kb plasmid, which allowed us to normalize the band sizes.

### 2.5. Detection of Antibiotic Resistance-Associated Genes

The presence of genes related to the main β-lactamase enzymes that confer resistance to broad-spectrum β-lactams was detected; likewise, the presence of three DNA fragments associated with resistance to fluoroquinolones was identified. The genes associated with resistance to β-lactams were as follows: *bla*_CTX-M-1 and 8_, *bla*_CTX-M-9_, *bla*_CTX-M-2_, *bla*_CTX-M-15_, *bla*_TEM_, and *bla*_SHV_. Additionally, those associated with resistance to fluoroquinolones and aminoglycosides were *qepA*, *aac(6′)-Ib* (Also related with cross-resistance to aminoglycosides) and *qnrB*. The oligonucleotide sequences and conditions were previously reported (Table S1) [32,33,34,35,36]. PCR products were resolved in a 2% agarose gel and stained with GelStar (Lonza, Morrinstown, NJ, USA).

### 2.6. Extended-Spectrum β-Lactamase (ESBL)-Producing Phenotype

Previously characterized cefotaxime (CTX)-, ceftazidime (CFZ)-, ceftriaxone (CRO)-, aztreonam (ATM)-, or cefepime (FEP)-resistant isolates [16] were selected to evaluate their ability to produce ESBL. Determination of the ESBL phenotype was performed by the double-disk diffusion method, using clavulanic acid as an inhibitor. For each suspected isolate, a bacterial suspension adjusted to 0.5 on the McFarland scale was prepared in Müeller-Hinton broth; Müeller-Hinton agar plates were inoculated with the adjusted suspensions with a sterile swab, allowed to dry for 5 min, and the antibiotics discs to be evaluated were placed. The discs were placed at 1.5 cm from the inhibitor center to center. The presence of an inhibition halo, indicative of synergy, between the inhibitor and any of the β-lactams evaluated was considered positive for the phenotype. Additionally, negative results were confirmed following the guidelines established by CLSI [37] for the determination of ESBL phenotypes in enterobacterales; for this purpose, Müeller-Hinton agar plates were inoculated with the isolates and discs of each β-lactam to be evaluated were placed in the presence and absence of 10 µg of clavulanic acid, with a difference of 5 mm in inhibition halo diameter between antibiotics with clavulanate and without clavulanate indicating a positive ESBL phenotype.

### 2.7. Carbapenemase-Producing Phenotype

For the detection of enzymes with carbapenemase activity, only isolates resistant to carbapenems were selected [16]. This detection was performed following the criteria established by CLSI [37] using the modified carbapenems inactivation method (mCIM); for this purpose, tubes of trypticase soy broth (TSB) broth were inoculated with the isolate in question, and the suspension was homogenized with a vortex for 10–15 s; with sterile forceps, a single disc of carbapenem (10 µg) was immersed in the bacterial suspension and incubated at 37 °C for 4 h. Subsequently, a 0.5 McFarland’s adjusted suspension of *E. coli* strain ATCC 25922 (carbapenem sensitive) was prepared in a sterile saline solution. The adjusted inoculum was mass seeded with a sterile swab on a Müeller-Hinton agar plate and allowed to dry for 10 min. The carbapenem discs were extracted from the problem strain inoculum, drained, and plated onto the MH agar plate previously inoculated with *E. coli* ATCC 25922. The plate was incubated at 37 °C for 18–24 h, and the results were interpreted according to CLSI, considering a difference of ≥5 mm between the hydrolyzed antibiotic vs. non-hydrolyzed antibiotic as a positive phenotype.

### 2.8. Bacterial Adherence Patterns to HeLa Cells

Adherence assays were performed in twenty clinical isolates according to previously reported protocols [16]. The cell lineage used was HeLa ATCC CCL-2, which is a cervix cancer cell line; cells were grown in culture plates with DMEM (Dulbecco’s Modified Eagle Medium, St. Louis, MO, USA), supplemented with 5% fetal bovine serum (D5F) (GIBCO, St. Louis, MO, USA), and incubated at 37 °C with 5% carbon dioxide (CO_2_) until sub-confluence of 70–90%. Once grown, the cells were washed with sterile phosphate-buffered solution (PBS) and trypsinized for two minutes to detach the monolayer. Subsequently, ten milliliters of fresh D5F were added and centrifuged for seven minutes at 500× *g* to remove trypsin and to obtain the cell pellet, the culture medium was decanted, and then 3 mL of D5F were added to resuspend the cells. One hundred microliters of the suspension were taken for cell counting in a hemocytometer chamber. Next, sterile coverslips were placed in each well of a six-well polystyrene plate with 2 mL of D5F, and a suspension of 50,000 HeLa cells was added. The plate was incubated for 24 h at 37 °C and 5% CO_2_ until a 70–90% confluence was reached.

A bacterial suspension adjusted to 0.5 on the McFarland scale (1 × 10^8^ bacteria per milliliter) was prepared from a 24 h pre-culture in Luria-Bertani broth (LB) of each clinical isolate to be evaluated; the adjustment was made in D5F broth without antibiotic. Once prepared, 15 µL of the adjusted inoculum was added to each well of the plate with HeLa cells (MOI: 30:1 Bacteria:Hela). The plate was incubated for three hours at 37 °C and 5% CO_2_. Then, the monolayer was washed with sterile PBS to remove unattached bacteria. This last procedure was performed three times. The cells were then fixed with methanol for ten minutes, allowed to dry at room temperature, and stained with Giemsa for fifteen minutes. The coverslips were removed and mounted on slides to count the adherent bacteria per HeLa cell. The adherence profile for each clinical isolate was also determined by lightfield microscopy with the 100× objective. *E. coli* EDL933 (EHEC), which is characterized by a localized adherence pattern, was used as a control strain.

### 2.9. Pathotypes Detection

The presence of genes related to specific DEC pathotypes was detected in twenty clinical isolates by PCR according to primer sequences and conditions previously reported [38,39,40,41] (Table S1). Hybrid pathotypes were classified as those isolates that simultaneously presented any of the UPEC associated virulence genes or PAIs in combination with any of the DEC pathotype-associated genes (*daaE*, *eaeA*, *bfpA*, pCVD432). The *daaE* gene is associated with diffuse adherent *E. coli* (DAEC) and diffuse adherent pattern, *eaeA* and *bfpA* are genes related to enteropathogenic *E. coli* (EPEC) and localized adherent pattern. In contrast, plasmid pCVD432 is associated with enteroaggregative *E. coli* (EAEC) and aggregative adherent patterns.

### 2.10. Multiplex PCR for Dominant Sequence Types (STs) in EXPEC Isolates

The sequence types (STs) ST131, ST69, and ST73 from clinical isolates were identified by multiplex PCR reactions according to previously reported oligonucleotides and conditions [42] (Table S1).

### 2.11. Enterobacteriaceae Repetitive Intergenic Consensus (ERIC) PCR

The clonal relationship was detected by amplification of ERIC (*Enterobacteriaceae* repetitive intergenic consensus) fragments by PCR (ERIC-PCR) using previously standardized oligonucleotides [43]. Each PCR reaction was performed using a mixture containing 2 µL of buffer, 0.5 µL of a mixture of dNTP (10 mM), 1.5 µL of MgCl_2_ (25 mM), 1 µL of each primer (20 µM), 0.1 µL of GoTaq^®^ Flexi DNA polymerase (PROMEGA, USA), 1.5 µL of template DNA (50–75 ng), and nuclease-free water until a final volume of 15.5 µL of reaction was obtained. The mixture was submitted to one cycle at 95 °C for five minutes, followed by 40 cycles at 95 °C for 1 min, 50 °C 1 min, 72 °C for 8 min, and one more cycle of 72 °C for 16 min. PCR products were resolved on 1.5% agarose gels and stained with GelStarTM (Lonza, Morristown, NJ, USA). Band profiles were analyzed using GelAnalyzer 19.1 software (available on http://www.gelanalyzer.com/?i=1; accessed on 1 June 2023) to determine the sizes of each product, and, subsequently, dendrograms were constructed by the unweighted paired-pairing by the arithmetic mean (UPGMA) method and the DICE algorithm to detect similarities between ERIC patterns, dendogram was constructed with iTOL 6.8 [44].

### 2.12. Statistical Analysis

For the statistical analysis, Pearson’s correlation test was implemented. Analysis was performed with JASP software version 0.16.1.

## 3. Results

### 3.1. Clinical Isolates of E. coli from UTI Cases in Mexico Harbor Pathogenicity Islands

The presence of PAIs was observed in 29 (72.5%) of the forty analyzed clinical isolates. One isolate presented six PAIs (isolate 26). Three of these isolates presented five of the identified PAIs; three showed only one PAI, and the other thirty-three isolates showed between two and four PAIs.

**Figure 1:**
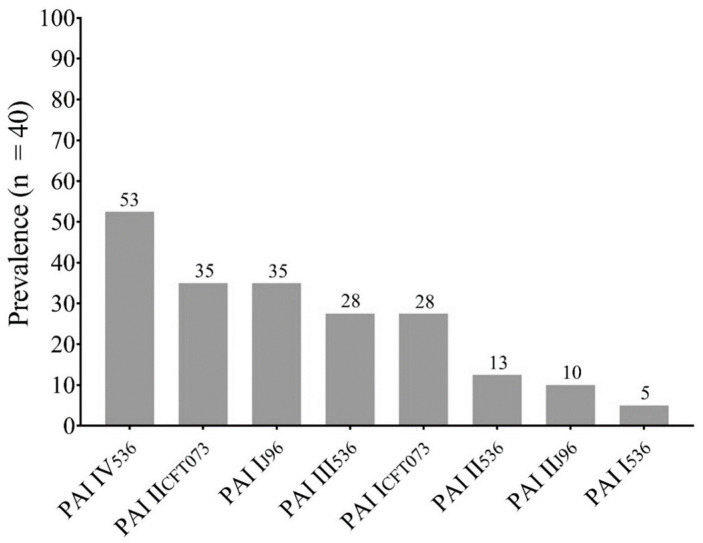
Prevalence of Pathogenicity Islands (PAIs) in This Study.

The most prevalent pathogenicity island was PAI IV_536_ (72.41%), followed by PAI II_CFT073_ (48.27%) and PAI I_J96_ (48.27%). The eight PAIs investigated were found in the *E. coli* strains analyzed (Figure 1).

The PAI profiles of each isolate are shown in Table 1. Statistical analysis was performed to detect a possible correlation between the co-occurrence of PAIs in the clinical isolates, and a strong positive correlation was observed in the co-occurrence of PAI I_536_–PAI I_J96_ (*r* = 0. 3126; *p* = 0.02), PAI I_536_–PAI I_CFT073_ (*r* = 0.3725; *p* = 0.009), PAI III_536_–PAI II_CFT073_ (*r* = 0.4871; *p* = 0.001), PAI IV_536_–PAI II_CFT073_ (*r* = 0.4881; *p* = 0.001), PAI I_J96_–PAI I_CFT073_ (*r* = 0.3698; *p* = 0.009), PAI I_536_–PAI II_J96_ (*r* = 0.3059; *p* = 0.02), and PAI II_J96_–PAI I_CFT073_ (*r* = 0.5412; *p* = <0.001).

### 3.2. Clinical Isolates of E. coli Are ESBL Producers

The antibiotic resistance profiles of the analyzed clinical isolates were previously reported [16] and are shown in Table S2. In total, 34 (85%) isolates were resistant to third or fourth generation cephalosporins or to the tested monobactam antibiotics; these 34 isolates were selected to detect the ESBL-producing phenotype. Twenty-two (65%) of the 34 selected clinical isolates exhibited an ESBL-producing phenotype. The produced enzymes hydrolyzed one to five of the evaluated antibiotics. The most frequent substrate profiles are shown in Table 2. Interestingly, five of the isolates (ID: 5, 27, 31, 35, and 36), despite being resistant to most of the β-lactam antibiotics tested (they were resistant to cefoxitin (CX), third generation cephalosporins, and at least one of the two β-lactams with inhibitors tested (amoxicillin-clavulanic acid (AMC) or ampicillin-sulbactam (AMS) did not show an ESBL-producing phenotype.

As expected, a statistical significance was observed in co-occurrence of ESBL phenotypes and resistance to β-lactam antibiotics, mainly between ESBL producers and resistance to CTX/CFZ (*r* = 0.49, *p* = 0.005), CRO (*r* = 0.41, *p* = 0.017), ATM (*r* = 0.46, *p* = 0.009); CFZ and CRO (*r* = 0.71, *p* = <0.001), FEP (*r* = 0.49, *p* = 0.005); FEP and ATM (*r* = 0.68, *p* = <0.001). On the other hand, when comparing virulence with resistance characteristics in this study, a negative correlation between positive ESBL phenotype for CTX and prevalence of PAI I_J96_ (*r* = −0.40, *p* = 0.05) and PAI IV_536_ (*r* = −0.28; *p* = 0.04) was observed.

### 3.3. Carbapenem Resistance Is Mediated by Carbapenemases Production

Five of the analyzed clinical isolates were resistant to carbapenems (ID: 7, 27, 35, 39, and 40, Table S2). These isolates were selected to detect the carbapenemase (CAR) production phenotype using the mCIM method previously described. Interestingly, all evaluated isolates were CAR positive, three for imipenem and two for meropenem. Two isolates (27 and 35) were classified previously as a non-producer ESBL phenotype with CX and AMC resistance, while two more isolates (39 and 40) were also ESBL producers, and the last clinical isolate (7) was resistant only to carbapenems (Table 2 and Table S2).

### 3.4. Clinical Isolates Harbored Antibiotic Resistance Genes

#### 3.4.1. β-Lactams

ESBL Producers, aminoglycosides, and fluoroquinolones-resistant isolates were selected. Thirty-two (80%) were resistant to amikacin or gentamicin, while only fifteen (38%) were fluoroquinolone-resistant (ciprofloxacin, levofloxacin, or norfloxacin) (Table S2).

Among twenty-two isolates with ESBL-producing phenotypes, we observed the enzyme-associated genes in twenty (91%) of them. The prevalence of each analyzed gene was as follows: *bla*_CTX-M-2_ (60%), *bla*_CTX-M-1 and 8_ (55%), *bla*_TEM_ (30%), *bla*_CTX-M-9_ (20%), and *bla*_CTX-M-15_ (10%). No positive isolates for the *bla*_SHV_ gene were observed.

On the other hand, despite showing resistance to β-lactams, twelve of the isolates did not exhibit a positive ESBL phenotype. However, they presented genes associated with these enzymes, mainly *bla*_TEM_ (seven isolates), *bla*_CTX-M-1 and 8_ (four isolates), *bla*_CTX-M-2_ (four isolates), *bla*_CTX-M-9_ (two isolates), and *bla*_CTX-M-15_ (one isolate). Interestingly, within this group of isolates are the five isolates that also showed resistance to cephamycins (cefoxitin) and at least one of the antibiotics with β-lactamase inhibitors (clavulanic acid or sulbactam). The remaining six isolates did not show resistance to the β-lactam antibiotics tested and did not have a production phenotype, but *bla*_CTX-M-1 and 8_ (three isolates), *bla*_TEM_ (three isolates), and *bla*_CTX-M-2_ (one isolate) were detected; this is probably due to non-expressed genes or non-functional proteins.

The statistical analysis showed a significance in the correlation between the prevalence of *bla*_CTX-M-2_ and ESBL phenotype for CFZ (*r* = 0.49, *p* = 0.005), CRO (*r* = 0.41, *p* = 0.02). Likewise, a positive correlation was observed for *bla*_CTX-M-15_ and CRO (*r* = 0.4, *p* = 0.03) and FEP (*r* = 0.4, *p* = 0.04). The co-occurrence of *bla*_CTX-M-9_ and *bla*_CTX-M-15_ was also statistically significant (*r* = 0.045, *p* = 0.009).

#### 3.4.2. Aminoglycosides or Fluoroquinolones

Thirty-two (80%) isolates were resistant to at least one of the tested aminoglycosides (AMK and GM, Table S2), while fifteen (38%) were resistant to at least one of the evaluated fluoroquinolones (CIP, LVX, and NOR). These isolates were selected to determine the prevalence of genes associated with resistance to these two families of antimicrobials.

Fourteen (93%) of the fifteen isolates resistant to fluoroquinolones showed the presence of at least one of the determined genes, with the acetylase associated gene *aac(6′)-Ib* (93%) being the most prevalent, followed by *qepA* (66%), associated with efflux pumps, and *qnrB* (60%) related to protective proteins of the target site of the antibiotic. On the other hand, the *aac(6′)-Ib* gene was also found in high prevalence among aminoglycoside-resistant isolates (54.2%). A strong positive correlation was observed in the prevalence of aminoglycoside vs. fluoroquinolone resistance and each resistance gene analyzed (*r* ≥ 0.99, *p* < 0.001, Table S3). Interestingly, a strong positive correlation between ESBL producer phenotypes, resistance to aminoglycosides, fluoroquinolones, and the presence of previously mentioned genes was also observed (*r* ≥ 0.99, *p* < 0.001) (Table S3).

Regarding virulence, no statistical significance was observed between the prevalence of PAI and the evaluated antibiotic-resistance genes.

### 3.5. Plasmids of Clinical Isolates Are Correlated with Resistance Genes Prevalence

The presence of plasmid bands was determined in the 40 analyzed clinical isolates. Only twenty-three (57.5%) of the isolates possessed plasmids. The number of bands per isolate ranged from one to eleven, with a mean of four plasmid bands per isolate. The interval band sizes observed, and their prevalence, were as follows: 1–5 kb (91.3%), 6–10 kb (39.1%), 11–20 kb (17.4%), 21–30 kb (9%), 31–50 kb (13%), and 101–120 kb (9%). No statistically significant was observed between the presence of plasmids vs. the virulence and resistance-evaluated features (*p* > 0.05) (Table S4). However, a strong positive correlation was found between the prevalence of 51–100 kb with resistance to FEP (*r* = 0.38, *p* = 0.01) and 101–120 kb with resistance to LVX (*r* = 0.33, *p* = 0.037), FEP (*r* = 0.69, *p* < 0.001), ATM (*r* = 0.4, *p* = 0.01), and IMP (*r* = 0.37, *p* = 0. 02) and the β-lactamase-producing phenotypes ESBL-FEP (*r* = 0.37, *p* = 0.02), ESBL-ATM (*r* = 0.54, *p* < 0.001), and *qepA* gene prevalence (*r* = 0.4, *p* = 0.01) (Table S5).

### 3.6. ST131 Is the Most Prevalent Clonal Group

Within the 40 analyzed isolates, only 30 (75%) showed the presence of molecular markers associated with the determined STs. The 60% of the isolates were classified as belonging to the ST131 clonal group, followed by the ST69 group (13%), and ST73 (3%). No isolates belonging to the ST95 group were observed.

No significant association was shown between the presence of PAI, and the ST groups identified. However, a statistically significant association was observed in the higher prevalence of resistance to fluoroquinolones (*p* = 0.05) in the ST131 clonal group (Table 3). In this group, we also showed a high prevalence of isolates with genes associated with antibiotic resistance, mainly *bla*_CTX-M-1 and 8_ (58.4%, *p* = 0.005), *bla*_CTX-M-9_ (17%, *p* = 0.02), *qepA* (33.3%, *p* = 0.001), *aac(6′)-Ib* (46%, *p* = 0.002), and *qnrB* (33.3%, *p* = <0.001). On the other hand, a strong positive correlation was shown in the higher prevalence of the *bla*_TEM_ gene (*r* = 0.46, *p* = 0.002) in the ST69 clonal group.

### 3.7. Clinical Isolates Show Mixed Adherence Patterns, including Some Associated with Diarrheagenic Pathotypes of E. coli

**Figure 2:**
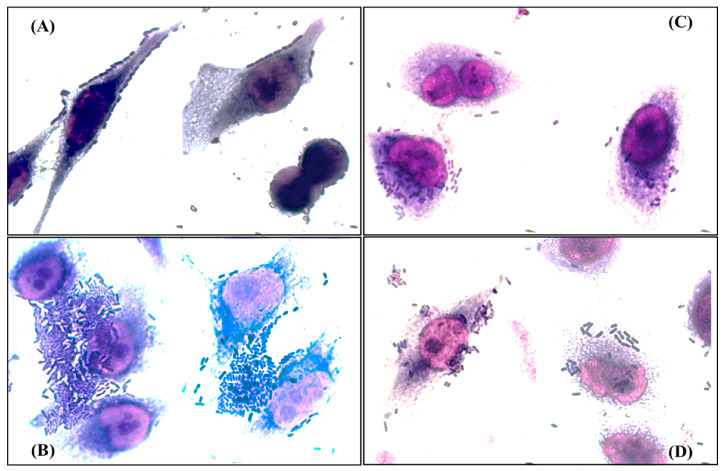
Adherence patterns detected in the analyzed clinical isolates. (**A**) Tandem bricks pattern; (**B**) aggregative pattern; (**C**) diffuse pattern; (**D**) localized pattern. Bright field microscopy 50× objective.

According to the profile of genes related to adherence of each strain [10], twenty isolates were randomly selected and their adherence patterns were determined. The patterns observed were bricks (85%), localized (55%), aggregative (20%), and diffuse (15%) (Figure 2). Interestingly, only eight (40%) of the isolates exhibited a single adherence pattern, six of them showed the tandem bricks pattern, while twelve (60%) exhibited mixed adherence patterns associated with different pathotypes of *E. coli*, and, of these twelve, pathogenicity islands related to prototype strains of uropathogenic *E. coli* were found in nine (77%) (Table 4 and Table S6 and Figure S1).

These findings and the presence of virulence factors, characteristic of DEC, in the clinical isolates suggest the presence of UPEC-DEC hybrid pathotypes in the studied bacterial population.

### 3.8. Clinical Isolates Have Molecular Markers Associated with DEC Pathotypes

Considering the adherence results, molecular markers associated with DEC pathotypes were determined for the 20 clinical isolates.

In 80% of the selected isolates, the *bfpA* gene was detected, the plasmid-associated gene pCVD432 was found in 30%, the *daaE* gene in 20%, and the *eaeA* gene in 5%. Four isolates did not present any of the DEC genes (Table 4). On the other hand, nine isolates presented in between two and four of the genes determined. The presence of the eaeA gene and the absence of *bfpA* is related to atypical EPEC. In this study, only one isolate harbored the eaeA gene, but this was also positive for *bfpA*. Interestingly, ten isolates harbored PAIs in addition to one or more pathotype-associated genes. These results and virulence factors related to UPEC in analyzed clinical isolates (Table S6) indicate the existence of hybrid clinical isolates of *E. coli* in the Mexican population with UTIs, including triple and quadruple hybrid strains (Table 4).

### 3.9. ERIC Fingerprint Pattern in Clinical Isolates

All analyzed isolates showed between 1 and 12 bands, with a higher prevalence of isolates with eight (38%) and six (20%) bands. The size interval of the bands was from 0.15 kb to 10 kb.

**Figure 3:**
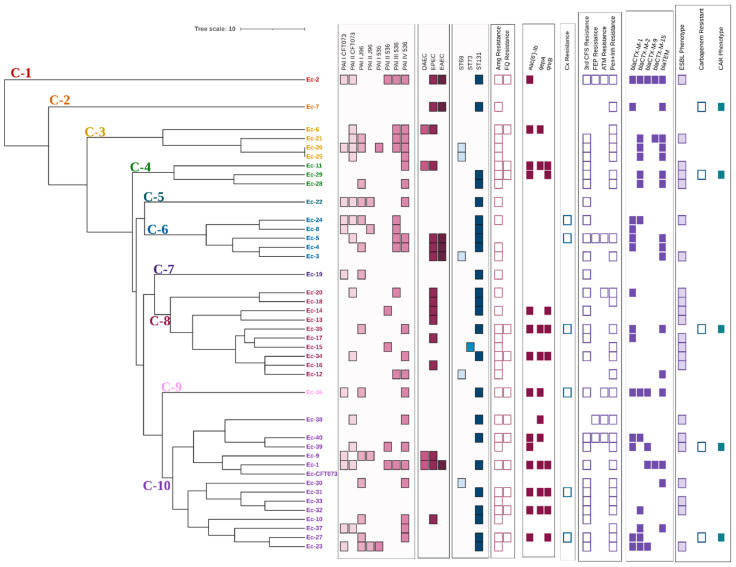
Dendrogram derived from the UPGMA pairing and the DICE coefficient representing ERIC-PCR patterns, PAI profile, DEC pathotype-associated genes, STs, and resistance characteristics of clinical *E. coli* isolates analyzed. PAI: Pathogenicity island; DAEC: Diffuse adherent *E. coli* (presence of *daaE* gene); EPEC: Enteropathogenic *E. coli* (presence of *bfpA* and *eaeA* genes); EAEC: Enteroaggregative *E. coli* (presence of pCVD432 gene); ST: Sequence types; Amg: Aminoglycosides; FQ: Fluoroquinolones; Cx: Cefoxitin; 3rd CFS: Third generation cephalosporins; FEP: Cefepime; ATM: Aztreonam; Pen+Inh: Penicillins with ESBL inhibitor; CAR: Carbapenemase.

According to the results obtained in the bands analysis and the dendogram generated by applying the UPGMA method and the DICE coefficient with a similarity value of 80%, the forty analyzed clinical isolates were distributed in ten different clusters (C-1–C-10) (Figure 3). Only two isolates (Ec-25 and Ec-26 presented the same ERIC profile; both isolates belonged to the ST69 group and did not present genes associated with other pathotypes different from UPEC. Regarding their virulence characteristics, differences were observed in the profile and number of PAI of each isolate; however, the remaining characteristics associated with resistance were the same.

Whether the presence of the different molecular markers of virulence or resistance and the phenotypes of resistance were associated with any of the different clusters obtained was investigated. A positive correlation with statistical significance was found between PAI III_536_ vs. clinical isolates belonging to cluster 6 (*r* = 0.44, *p* = 0.002), resistance to third generation cephalosporins (*r* = 0.27, *p* = 0.05), and the ESBL phenotype (*r* = 0.29, *p* = 0.04) vs. cluster 8. On the other hand, fluoroquinolone resistance (*r* = −0.28, *p* = 0.04) and the prevalence of PAI I_CFT073_ (*r* = −0.36, *p* = 0.01) and PAI III_536_ (*r* = 0.31, *p* = 0.03) were negatively correlated with clusters 6, 8, and 10, respectively.

## 4. Discussion

Urinary tract infections are the second leading cause of morbidity in Mexico. *E. coli* is the main etiological agent of this disease and possesses a wide variety of genetic virulence and resistance determinants that have allowed it to become a successful pathogen. These genetic characteristics are commonly harbored in pathogenicity islands or plasmids that can be transferred, which promotes the spreading of these important characteristics.

In this work, the presence of molecular markers associated with pathogenicity islands was determined in 40 clinical isolates of *E. coli* obtained from Mexican women with UTIs. Previously, these isolates had been classified as highly virulent with important characteristics for the development of both lower and upper UTIs, and a co-occurrence of genes that are commonly found harbored in pathogenicity islands was also observed [16].

Therefore, in this work we determined the presence of these elements in the previously mentioned isolates. Upon analysis, we found that 72.5% of them presented PAIs associated with UPEC, with a high prevalence of PAI IV of *E. coli* 536 (53%), followed by PAI II_CFT073_ (35%) and PAI I_J96_ (35%). It is important to mention that we only determined the presence of eight pathogenicity islands; however, there are others, such as those of UPEC UMN026 (5 PAIs), UPEC UTI 89 (8 PAIs), five more of UPEC 536 and 11 more of UPEC CFT073 [12], that were not identified in this study and that could probably also explain this co-occurrence of virulence-associated genes that we previously reported.

The PAI IV of *E. coli* 536, also known as high pathogenicity island (HPI), is one of the most stable islands reported in *E. coli* and is thought to be fixed in the chromosome [11,45], probably being one of the first PAIs to be acquired, which could explain its high prevalence in this and other previously reported studies, including in commensal strains, which has led to the assumption that, rather than a pathogenicity island, it could be a fitness island; however, its content of virulence-associated genes makes it an important marker of pathogenicity [9,46].

On the other hand, PAI I of *E. coli* 536 was the less prevalent, and it has been reported that this island together with PAI II_536_ and PAI III_536_ present higher instability. Interestingly, conditions such as the presence of antimicrobials, such as nitrofurantoin, gentamicin, cotrimoxazole, ampicillin and fosfomycin, all implemented in the treatment of UTIs in Mexico, induce the expression of integrases on this MGE and their cleavage [47], which could explain the lower prevalence of these PAIs in our study.

The prevalence of clinical isolates with PAIs found in this study is higher than previously reported in another work performed in Sonora and Puebla, which until now was the only report of these specific pathogenicity islands in our country; however, the prevalence of the PAI of *E. coli* 536 had not been previously described [18]. The prevalence of PAI IV_536_ and the other determined islands are similar to those reported by Sabaté in 2006 in clinical isolates of UPEC; however, Sabaté and other authors did not report the presence of *E. coli* J96 PAIs [9,11,46,48], which could indicate an association to the geographic area or patient group, since, in this work and in previous reports regarding Mexico, both islands were observed in adult females [18], whereas reports in other countries include the male and pediatric populations.

The PAI of *E. coli* 536 harbors genes related to upper UTIs, including toxins such as alpha hemolysin and its gene operon (*hlyA-D*) (PAI I), the pyelonephritis-associated pilus operon (*papA-F*) (PAI II), S-type fimbriae (*sfa*) and its gene operon, iron scavengers such as salmochelin and its gene operon (PAI III), yersiniabactin-related genes (PAI IV) [49], and genes associated with capsule (*kps*) production (PAI V) [50]. This, together with the high prevalence of the PAI of *E. coli* CFT073 (an isolate characterized as pyelonephritic), the PAI of *E. coli* J96, and their co-occurrence confirm the great genomic plasticity of *E. coli* and the elevated pathogenic potential of the analyzed clinical isolates. Interestingly, despite its importance, this is the second article in our country that reports the presence of these genetic elements in clinical isolates of *E. coli* obtained from patients with UTIs.

In addition to its virulence and its evident impact on the development of UTIs, *E. coli* possesses a wide variety of antibiotic resistance determinants. In this regard, β-lactam antibiotics, fluoroquinolones, and aminoglycosides represent three of the categories that, depending on patient characteristics, are widely employed in UTI therapeutics. Recently, our working group published a systematic review in which a high prevalence of resistance to these groups of antimicrobials was observed in Mexico, with fluoroquinolones (58%) having the highest resistance, followed by β-lactam antibiotics (second to fourth generation cephalosporins: 35–57%) and aminoglycosides (26%) [6].

Resistance to this group of drugs is harbored mainly in MGEs such as plasmids and, in the case of β-lactams, is mediated by genes encoding for extended-spectrum β-lactamases (ESBL) [51]. In this study, 40 clinical isolates were analyzed, and 34 showed resistance to third- and fourth-generation cephalosporins or monobactams. Among these, 22 strains (65%) exhibited an ESBL-producing phenotype and 20 isolates contained genes associated with β-lactamases. The bla_CTX-M-2_ gene was the most prevalent (60%), followed by bla_CTX-M-1_ (55%) and bla_TEM_ (30%). These results align with other studies in our country regarding the prevalence of bla_CTX-M-1_ and bla_TEM_ [52,53,54]. However, the frequency of blaCTX-M-2 in our study was significantly higher than previously reported in Mexico [55].

Likewise, a high prevalence of the *bla*_CTX-M-15_ gene (33–96%) has been observed in other Mexican states, such as Mexico City and Jalisco [56,57,58], associated with the ST131 clone. Here, we observed a reduced prevalence of this gene compared to those studies (10%), even though 70% of our isolates were molecularly identified as belonging to the ST131 clonal group, which could indicate a probable association with the geographical area or patient group.

Five of the analyzed isolates showed resistance to third generation cephalosporins and monobactam aztreonam, and one strain was also resistant to cefepime. Interestingly, these isolates did not present an ESBL-producing phenotype, but their susceptibility profile showed resistance to cephamycins and combined antibiotics (amoxicillin-clavulanic acid and/or ampicillin-sulbactam). This resistance profile is commonly associated with pathogenic AmpC β-lactamase producers, mainly those harbored in plasmids (pAmpC), which, although in *E. coli* are infrequent, their existence has been reported in clinical isolates obtained from patients with UTIs in other countries [59,60]. In Mexico, there are reports of genes associated with these pAmpC in clinical isolates of UPEC, with CMY (mainly CMY 1, 2, and 23) being the most prevalent [61].

The potential presence of pAmpC in the clinical isolates analyzed is significant due to the complications it can cause in treating infections. Its presence is associated with therapeutic failures, and its plasmid location increases the likelihood of dispersion. Therefore, identifying molecular markers associated with plasmidic AmpC enzymes in these clinical isolates would be both interesting and epidemiologically important.

Regarding resistance to aminoglycosides and quinolones, this was observed in 80% and 38%, respectively, of the analyzed isolates. The occurrence of these phenotypes was coincident with the presence of genes associated with resistance to these groups of antimicrobials, with a high prevalence of the *aac(6′)-Ib* gene (93%), which is related to cross-resistance to fluoroquinolones and aminoglycosides [62,63], followed by *qepA* (60%) and *qnrB* (30%).

The *aac(6′)-Ib* gene is also localized in integrons, mainly class 1 integrons, which are present in plasmids Raherison [63]. Therefore, and considering the high prevalence of resistance to fluoroquinolones in our country (which exceeds 50% according to the reports available until 2021 [18]), it would be interesting to determine the presence of these elements in the analyzed strain.

A strong positive correlation was observed between ESBL production with each of the determined genes and the resistance phenotypes to aminoglycosides and fluoroquinolones; this could be because these genes and most of those associated with ESBL are commonly harbored in plasmids, and there are reports of their co-existence [64,65]. In addition, when analyzing the plasmid profiles, a strong positive correlation was found between each resistance gene and phenotypes determined with plasmid bands of variable sizes. This reinforces our hypothesis regarding the co-occurrence of these characteristics in the population analyzed; however, further studies are needed to confirm it since many of the observed bands could be plasmid isoforms.

In addition, it was observed that five of the isolates were carbapenemase producers, and, due to their resistance profiles and the number of antibiotic categories to which they are resistant, they were previously classified as multidrug-resistant (Table S2). This result is alarming, considering that carbapenems are implemented as a last resource in the treatment of infectious processes and reflect the urgency of searching for therapeutic alternatives.

On the other hand, 60% of the analyzed isolates in this study belong to the ST131 group. This clonal group of *E. coli* is globally distributed and is important due to its significant genetic content associated with virulence and resistance. It has been reported that ST131 strains are predominantly non-susceptible to fluoroquinolones in addition to presenting co-resistance to antimicrobials such as aminoglycosides, co-trimoxazole, and β-lactams [66,67]. In our study, isolates from the ST131 clonal group showed higher resistance to all evaluated antibiotic categories. However, only resistance to fluoroquinolones was statistically correlated.

In the same way, the genes associated with ESBL, except for *bla*_TEM_ and all those related to fluoroquinolones and aminoglycoside resistance, were more prevalent in this clonal group. Our results agree with those previously reported in countries such as Turkey [68] concerning the association of *bla*_CTX-M-1_ and *bla*_CTX-M-15_ with the ST131 group. However, we observed a low prevalence of *bla*_CTX-M-15_ (10%) in the analyzed isolates, which differs from that reported in Turkey (38%), in India by Hussain (100%) [69], and in other states of Mexico by Reyna-Flores (97%). These results suggest that the prevalence of *bla*_CTX-M-15_ is more influenced by the geographical area and the environment to which the pathogen is exposed than the clonal group to which they belong, as well as that, considering the higher prevalence of *bla*_CTX-M-2_ (60%), as well as the prevalence of *bla*_CTX-M-9_ (55%) and *bla*_TEM_ (30%), the ST131 clonal group presents a greater diversity of genes associated with β-lactam resistance than that reported in the previously mentioned works.

Finally, the existence of *E. coli* isolates that possess virulence determinants of more than one pathotype has been previously described in other countries [70,71]; these strains are considered hypervirulent and represent an important health risk. In this study, we determined the presence of molecular markers associated with intestinal pathotypes of *E. coli*; the genes were selected according to the observed adherence profiles. There is no characteristic adherence pattern for UPEC; however, in previous studies in Mexico, it has been reported that some clinical isolates of UPEC can adopt an adherence pattern that has been called “train wagon” [72] in HeLa cells, which is like the one we observed and identified as the tandem bricks pattern.

Of the 20 isolates analyzed, genetic determinants of intestinal pathotypes were found in 16 (80%), being mainly the *bfpA* gene, which is associated with the localized adherence pattern and enteropathogenic *E. coli*. This result, together with the existence of UPEC virulence factors such as P-type pili (*papC*, *papG-II*), S-type pili (*sfa*), aerobactin (*iucD*, *iutA*), yersiniabactin (*fyuA*), capsular antigens (*kpsM*), and vacuolizing autotransporter toxin (*vat*) that were previously reported and that are considered as molecular markers of ExPEC-UPEC [73], in addition to the presence of pathogenicity islands associated with UPEC in these isolates, confirm the existence of hybrid pathotypes in the Mexican population with UTIs, so it would be important to determine their implications in the clinical treatment and diagnosis of the disease.

## 5. Conclusions

The clinical isolates analyzed showed virulence characteristics attributed to intestinal pathotypes and UPEC, which demonstrates the presence of hybrid pathotypes (UPEC/DEC) of *E. coli* causing urinary tract infections in our country. To our knowledge, this is the first article reporting the existence of UPEC/DEC hybrid pathotypes in Mexico. These isolates belong mainly to the ST131 clonal group and harbor a considerable number of genes associated with resistance to fluoroquinolones, aminoglycosides, and β-lactams, which are antibiotics widely employed for the treatment of UTIs. Furthermore, the high prevalence of ESBL-producing strains in our study and the presence of potential pAmpC- or carbapenemase-producing isolates is alarming because of their impact on antimicrobial therapy.

## Figures and Tables

**Table 1:** Pathogenicity Island (PAI) Profile in each Clinical Isolate of *E. coli* from ITU.

PAI Profile	Isolate ID
PAI I_536_, PAI I_J96_, PAI II_J96_, PAI I_CFT073_	23
PAI I_536_, PAI III_536_, PAIV_536_, PAI_J96_, PAI I_CFT073_, PAI II_CF073_	26
PAI I_J96_, PAI I_CFT073_	19
PAI I_J96_, PAII_J96_, PAI I_CFT073_	9
PAI II_536_	14,15
PAI II_536_, PAI III_536_, PAI IV_536_, PAI I_CFT073_, PAI II_CFT073_	1, 2
PAI II_536_, PAI IV_536_, PAI II_CFT073_	39
PAI III_536_, PAI II_CFT073_	20
PAI III_536_, PAI II_J96_, PAI I_CFT073_	8
PAI III_536_, PAI IV_536_	12
PAI III_536_, PAI IV_536_, PAI I_J96_, PAI II_CFT073_	21
PAI III_536_, PAI IV_536_, PAI II_CFT073_	5,6
PAI III_536_, PAI IV_536_, PAI_J96_	4
PAI III_536_, PAI_J96_, PAI I_CFT073_, PAI II_CFT073_	24
PAI IV_536_, PAI II_CFT073_	34
PAI IV_536_	11
PAI IV_536_, PAI I_CFT073_, PAI II_CFT073_	37
PAI IV_536_, PAI I_J96_	10, 27, 28, 30, 35
PAI IV_536_, PAI I_J96_, PAI I_CFT073_	36
PAI IV_536_, PAI I_J96_, PAI II_J96_, PAI I_CFT073_, PAI II_CFT073_	22
PAI IV_536_, PAI II_CFT073_	25, 38
Negatives	3, 4, 7, 13, 16–18, 29, 31–33, 40

PAI: Pathogenicity islands.

**Table 2:** ESBL Phenotype and ESBL Profiles in Respect to β-lactam Resistance Phenotype.

ID	Antibiotic Resistance								ESBL Phenotype					ESBL Profile
	CX	AMC	AMS	CFZ	CTX	CRO	FEP	ATM	CTX	CFZ	CRO	FEP	ATM	
1		R	R		R									
5	R	R	R	R	R	R	R	R						
10				R	R									
12		R	R		R									
19		R			R									
22				R	R									
25		R			R	R								
26		R	R		R									
27	R	R		R	R	R		R						
31	R	R		R	R	R								
35	R	R		R	R	R								
36	R	R		R	R	R		R						
21		R	R	R	R	R				+	+			CFZ, CRO
3		R	R		R	R					+			CRO
11		R		R	R				+					CTX
13					R			R	+					CTX
14					R	R			+					CTX
15		R			R				+					CTX
16		R			R				+					CTX
17					R				+					CTX
18		R	R		R				+					CTX
23		R			R	R			+					CTX
20		R			R	R		R	+				+	CTX, ATM
40		R	R	R	R	R	R	R	+				+	CTX, ATM
24	R	R		R	R	R			+	+				CTX, CFZ
30		R	R	R	R				+	+				CTX, CFZ
28		R	R	R	R	R			+	+	+			CTX, CFZ, CRO
32		R	R	R	R	R			+	+	+			CTX, CFZ, CRO
34		R	R	R	R	R			+	+	+			CTX, CFZ, CRO
2		R	R	R	R	R	R	R	+	+	+	+	+	CTX, CFZ, CRO, FEP, ATM
29		R		R	R	R		R	+	+	+	+	+	CTX, CFZ, CRO, FEP, ATM
39			R	R	R	R			+		+			CTX, CRO
33		R	R		R	R		R	+		+		+	CTX, CRO, ATM
38		R	R				R	R				+	+	FEP, ATM

Predetermined resistance profile [10]. R: Resistant; Blank space: Negative ESBL Phenotype; +: Positive ESBL Phenotype; CX: Cephamycin; AMC: Amoxicillin-Clavulanic Acid; AMS: Ampicillin-Sulbactam; CTX: Cefotaxime; CFZ: Ceftazidime; CRO: Ceftriaxone; FEP: Cefepime; ATM: Aztreonam. In underlined, CX and AMC or AMS resistant isolates without ESBL phenotype are shown. Only results for β-lactam Resistant isolates is shown.

**Table 3:** Prevalence of Virulence and Resistance Determinants by Clonal Group.

Feature	ST73 % (*n* = 1)	ST69 % (*n* = 5)	STNT % (*n* = 10)	ST131 % (*n* = 24)	*p* Value
PAI III_536_	0	40 (2)	20 (2)	29.2 (7)	
PAI IV_536_	0	80 (4)	50 (5)	50 (12)	
PAI II_CFT073_	0	40 (2)	40 (4)	33.3 (8)	
PAI II_536_	100 (1)	0	10 (1)	13 (3)	
PAI I_J96_	0	40 (2)	20 (2)	42 (10)	
PAI I_CFT073_	0	20 (1)	20 (2)	33.3 (8)	
PAI I_536_	0	20 (1)	0	4.2 (1)	
PAI II_J96_	0	0	10 (1)	13 (3)	
Aminoglycosides	100 (1)	80 (4)	70 (7)	83.3 (20)	
Fluoroquinolones	0	0	30 (3)	**50 (12)**	0.05
Cotrimoxazole	100 (1)	60 (3)	40 (4)	46 (11)	
Cephamycins	0	0	0	25 (6)	
3rd Cephalosporins	100 (1)	100 (5)	70 (7)	83.3 (20)	
4th Cephalosporins	0	0	0	17 (4)	
Monobactams	0	0	20 (2)	33.3 (8)	
Pen-Inh	100 (1)	100 (5)	70 (7)	79.2 (19)	
Carbapenems	0	0	10 (1)	17 (4)	
ESBL Phenotype	100 (1)	40 (2)	70 (7)	50 (12)	
CAR Phenotype	0	0	10 (1)	17 (4)	
*bla*_CTX m 1 y 8_	0	0	40 (4)	**58.3 (14)**	0.005
*bla*_CTX-M-9_	0	0	10 (1)	**17 (4)**	0.02
*bla*_TEM_	0	**100 (5)**	20 (2)	38 (9)	
*bla*_CTX-M-2_	100 (1)	60 (3)	30 (3)	46 (11)	
*bla*_CTX-M-15_	0	0	10 (1)	**8.3 (2)**	0.05
*qepA*	0	0	20 (2)	**33.3 (8)**	0.001
*aac(6′)-Ib*	0	0	30 (3)	**46 (11)**	0.002
*qnrB*	0	0	10 (1)	**33.3 (8)**	<0.001

PAI: Pathogenicity island; 3rd Cephalosporins: third generation cephalosporins; 4th Cephalosporins: fourth generation cephalosporins; Pen-Inh: penicillin’s combined with β-lactamases inhibitors; ESBL: β-lactamase positive phenotype; CAR: carbapenemase positive phenotype; *bla*: β-lactamase associated genes; *qepA*: efflux pump associated with fluoroquinolone resistance; *aac(6′)-Ib*: acetylase associated gene; *qnrB*: protective proteins associated with fluoroquinolone resistance; NT: non typable. The *p* values were obtained by Fisher Exact Test.

**Table 4:** Adherence Patterns, Pathotype-Associated Genes, PAI Profile and Potential Hybrid Pathotypes of Clinical Isolates.

ID	Pattern	Profile	PAI Profile	Pathotype
1	Bs/Lo	pCVD432, *eaeA*, *daaE*, *bfpA*	PAI III_536_, PAI IV_536_, PAI II_CFT073_, PAI II_536_, PAI I_CFT073_	UPEC/EPEC/DAEC/EAEC
2	Bs/Dif	pCVD432, *bfpA*	PAI III_536_, PAI IV_536_, PAI II_CFT073_, PAI II_536_, PAI I_CFT073_	UPEC/EAEC/EPEC
3	Bs/Lo/Ag	pCVD432, *bfpA*	-	UPEC/EAEC/EPEC
4	Bs/Lo	pCVD432, *bfpA*	PAI III_536_, PAI IV_536_, PAI_IJ96_	UPEC/EAEC/EPEC
5	Ag	pCVD432, *bfpA*	PAI III_536_, PAI IV_536_, PAI II_CFT073_	UPEC/EAEC/EPEC
6	Bs/Lo/Di	*daaE*, *bfpA*	PAI III_536_, PAI IV_536_, PAI II_CFT073_	UPEC/DAEC/EPEC
7	Bs	pCVD432, *bfpA*	-	UPEC/EAEC/EPEC
8	Bs/Lo	-	PAI III_536_, PAI I_CFT073_, PAI II_J96_	UPEC
9	Bs/Ag	*daaE*, *bfpA*	PAI I_J96_, PAI I_CFT073_, PAI II_J96_	UPEC/DAEC/EPEC
10	Bs/Lo	*bfpA*	PAI IV_536_, PAI_IJ96_	UPEC/EPEC
11	Bs	*daaE*, *bfpA*	PAI IV_536_	UPEC/DAEC/EPEC
12	Lo	-	PAI III_536_, PAI IV_536_	UPEC
13	Ag/Lo	*bfpA*	-	UPEC/EPEC
14	Bs	*bfpA*	PAI II_536_	UPEC/EPEC
15	Bs	-	PAI II_536_	UPEC
16	Ag/Bs/Lo	*bfpA*	-	UPEC/EPEC
17	Bs	*bfpA*	-	UPEC/EPEC
18	Bs	*bfpA*	-	UPEC/EPEC
19	Bs/Ag/Lo	-	PAI I_J96_, PAI I_CFT073_	UPEC
20	Bs/Lo/Di	*bfpA*	PAI III_536_, PAI II_CFT073_	UPEC/EPEC

ID: isolate; PAI: pathogenicity islands; Bs: tandem bricks pattern; Lo: localized pattern; Di: diffuse pattern; Ag: aggregative pattern.

## Data Availability

Data are contained within the article and Supplementary Materials.

## Supplementary Materials

The following supporting information can be downloaded at: https://www.mdpi.com/article/10.3390/cimb46060353/s1, Table S1: Oligonucleotide sequences and conditions used in this study; Table S2: Previously Reported Resistance Profiles of Analyzed Clinical Isolates; Table S3: Correlation Between Antibiotic Resistance Phenotypes and Genotypes; Table S4: Statistical Analysis for Association Between Plasmids Presence and Virulence or Resistance-Evaluated Features; Table S5: Statistical Analysis for Correlation Between Plasmid Sizes and Resistance or Virulence Features; Table S6: Virulence Genes, PAI Profiles, DEC Genes and Adherence Patterns of UPEC Clinical Isolates; Figure S1: Mixed Adherence Pattern in Clinical Isolates of *E. coli*. (**A**) Ec-9, shown a mixed aggregative and stacked-brick patterns; (**B**) Ec-2, shown a mixed diffuse adherence and stacked-brick patterns; (**C**) Ec-16, showing a mixed aggregative, diffuse adherence, and stacked-brick patterns; (**D**) Ec-16, shown an aggregative, localized, and steacked-brick patterns; (**E**) Ec-19, shown an aggregative, localized, and sticked-brick patterns. Yellow arrow indicated an aggregative pattern; blue arrows are for a stacked-brick pattern; green arrows show the localized pattern; red arrows indicate the diffuse adherence pattern.

## References

[1] Croxen M.A., Law R.J., Scholz R., Keeney K.M., Wlodarska M., Finlay B.B. (2013). Recent Advances in Understanding Enteric Pathogenic *Escherichia coli*. Clin. Microbiol. Rev..

[2] Russo T.A., Johnson J.R. (2000). Proposal for a New Inclusive Designation for Extraintestinal Pathogenic Isolates of *Escherichia coli*: ExPEC. J. Infect. Dis..

[3] Flores-Mireles A.L., Walker J.N., Caparon M., Hultgren S.J. (2015). Urinary Tract Infections: Epidemiology, Mechanisms of Infection and Treatment Options. Nat. Rev. Microbiol..

[4] Sharma K., Verma R. (2023). Riya Verma Urinary Tract Infections: A Review. World J. Biol. Pharm. Health Sci..

[5] Zhou Y., Zhou Z., Zheng L., Gong Z., Li Y., Jin Y., Huang Y., Chi M. (2023). Urinary Tract Infections Caused by Uropathogenic *Escherichia coli*: Mechanisms of Infection and Treatment Options. Int. J. Mol. Sci..

[6] Ballesteros-Monrreal M.G., Mendez-Pfeiffer P., Barrios-Villa E., Arenas-Hernández M.M.P., Enciso-Martínez Y., Sepúlveda-Moreno C.O., Bolado-Martínez E., Valencia D. (2023). Uropathogenic *Escherichia coli* in Mexico, an Overview of Virulence and Resistance Determinants: Systematic Review and Meta-Analysis. Arch. Med. Res..

[7] Mancuso G., Midiri A., Gerace E., Marra M., Zummo S., Biondo C. (2023). Urinary Tract Infections: The Current Scenario and Future Prospects. Pathogens.

[8] Gyles C., Boerlin P. (2014). Horizontally Transferred Genetic Elements and Their Role in Pathogenesis of Bacterial Disease. Vet. Pathol..

[9] Samei A., Haghi F., Zeighami H. (2016). Distribution of Pathogenicity Island Markers in Commensal and Uropathogenic *Escherichia coli* Isolates. Folia Microbiol..

[10] Schmidt H., Hensel M. (2004). Pathogenicity Islands in Bacterial Pathogenesis. Clin. Microbiol. Rev..

[11] Sabate M., Moreno E., Perez T., Andreu A., Prats G. (2006). Pathogenicity Island Markers in Commensal and Uropathogenic *Escherichia coli* Isolates. Clin. Microbiol. Infect..

[12] Desvaux M., Dalmasso G., Beyrouthy R., Barnich N., Delmas J., Bonnet R. (2020). Pathogenicity Factors of Genomic Islands in Intestinal and Extraintestinal *Escherichia coli*. Front. Microbiol..

[13] Sarshar M., Scribano D., Limongi D., Zagaglia C., Palamara A.T., Ambrosi C. (2022). Adaptive Strategies of Uropathogenic *Escherichia coli* CFT073: From Growth in Lab Media to Virulence during Host Cell Adhesion. Int. Microbiol..

[14] Zakaria A.S., Edward E.A., Mohamed N.M. (2022). Pathogenicity Islands in Uropathogenic *Escherichia coli* Clinical Isolate of the Globally Disseminated O25:H4-ST131 Pandemic Clonal Lineage: First Report from Egypt. Antibiotics.

[15] Chittò M., Berger M., Berger P., Klotz L., Dröge P., Dobrindt U. (2020). IHF Stabilizes Pathogenicity Island I of Uropathogenic *Escherichia coli* Strain 536 by Attenuating Integrase I Promoter Activity. Sci. Rep..

[16] Ballesteros-Monrreal M.G., Arenas-Hernández M.M.P., Barrios-Villa E., Juarez J., Álvarez-Ainza M.L., Taboada P., De la Rosa-López R., Bolado-Martínez E., Valencia D. (2021). Bacterial Morphotypes as Important Trait for Uropathogenic *E. coli* Diagnostic; a Virulence-Phenotype-Phylogeny Study. Microorganisms.

[17] Amábile-Cuevas C.F. (2010). Antibiotic Resistance in Mexico: A Brief Overview of the Current Status and Its Causes. J. Infect. Dev. Ctries.

[18] Ballesteros-Monrreal M.G., Arenas-Hernández M.M.P., Enciso-Martínez Y., Martinez de la Peña C.F., del C Rocha-Gracia R., Lozano-Zarain P., Navarro-Ocaña A., Martínez-Laguna Y., de la Rosa-López R. (2020). Virulence and Resistance Determinants of Uropathogenic *Escherichia coli* Strains Isolated from Pregnant and Non-Pregnant Women from Two States in Mexico. Infect. Drug Resist..

[19] Galindo-méndez M. (2019). Reservoirs of CTX-M Extended Spectrum β-Lactamase-Producing Enterobacteriaceae in Oaxaca. J. Microbiol. Exp..

[20] Gonçalves L.F., De Oliveira Martins P., De Melo A.B.F., Da Silva R.C.R.M., De Paulo Martins V., Pitondo-Silva A., De Campos T.A. (2016). Multidrug Resistance Dissemination by Extended-Spectrum β-Lactamase-Producing *Escherichia coli* Causing Community-Acquired Urinary Tract Infection in the Central-Western Region, Brazil. J. Glob. Antimicrob. Resist..

[21] Miranda-Estrada L.I., Ruíz-Rosas M., Molina-López J., Parra-Rojas I., González-Villalobos E., Castro-Alarcón N. (2017). Relación Entre Factores de Virulencia, Resistencia a Antibióticos y Los Grupos Filogenéticos de *Escherichia coli* Uropatógena En Dos Localidades de México. Enferm. Infecc. Microbiol. Clin..

[22] Zavala-cerna M.G., Segura-cobos M., Gonzalez R., Zavala-trujillo I.G., Navarro-perez S.F., Rueda-cruz J.A., Satoscoy-tovar F.A. (2020). The Clinical Significance of High Antimicrobial Resistance in Community-Acquired Urinary Tract Infections. Can. J. Infect. Dis. Med. Microbiol..

[23] Lagunas-Rangel F.A. (2018). Antimicrobial Susceptibility Profiles of Bacteria Causing Urinary Tract Infections in Mexico: Single-Centre Experience with 10 Years of Results. J. Glob. Antimicrob. Resist..

[24] Basu S., Mukherjee M. (2018). Incidence and Risk of Co-Transmission of Plasmid-Mediated Quinolone Resistance and Extended-Spectrum β-Lactamase Genes in Fluoroquinolone-Resistant Uropathogenic *Escherichia coli*: A First Study from Kolkata, India. J. Glob. Antimicrob. Resist..

[25] Randall L.P., Cooles S.W., Osborn M.K., Piddock L.J.V., Woodward M.J. (2004). Antibiotic Resistance Genes, Integrons and Multiple Antibiotic Resistance in Thirty-Five Serotypes of Salmonella Enterica Isolated from Humans and Animals in the UK. J. Antimicrob. Chemother..

[26] Chávez-Jacobo V., Ramírez-Díaz M., Silva-Sánchez J., Cervantes C. (2015). Resistencia Bacteriana a Quinolonas: Determinantes Codificados en Plásmidos. REB. Rev. Educ. Bioquímica.

[27] Bielaszewska M., Schiller R., Lammers L., Bauwens A., Fruth A., Middendorf B., Schmidt M.A., Tarr P.I., Dobrindt U., Karch H. (2014). Heteropathogenic Virulence and Phylogeny Reveal Phased Pathogenic Metamorphosis in *Escherichia coli* O2: H6. EMBO Mol. Med..

[28] Méndez-Moreno E., Caporal-Hernandez L., Mendez-Pfeiffer P.A., Enciso-Martinez Y., De la Rosa López R., Valencia D., Arenas-Hernández M.M.P., Ballesteros-Monrreal M.G., Barrios-Villa E. (2022). Characterization of Diarreaghenic *Escherichia coli* Strains Isolated from Healthy Donors, Including a Triple Hybrid Strain. Antibiotics.

[29] Walker D.I., McQuillan J., Taiwo M., Parks R., Stenton C.A., Morgan H., Mowlem M.C., Lees D.N. (2017). A Highly Specific *Escherichia coli* QPCR and Its Comparison with Existing Methods for Environmental Waters. Water Res..

[30] Carreón E. (2019). Estudio Molecular de La Resistencia y Virulencia de Cepas de *Escherichia coli* Productoras de β-Lactamasas de Espectro Extendido Aisladas de Vegetales Crudos. Master’s Thesis.

[31] Sambrook J., Russel D.W. (2000). Molecular Cloning, 3-Volume Set: A Laboratory Manual.

[32] Eftekhar F., Seyedpour S.M. (2015). Prevalence of Qnr and Aac(6’)-Ib-Cr Genes in Clinical Isolates of *Klebsiella* Pneumoniae from Imam Hussein Hospital in Tehran. Iran. J. Med. Sci..

[33] Garza-González E., Bocanegra-Ibarias P., Bobadilla-del-Valle M., Ponce-de-León-Garduño L.A., Esteban-Kenel V., Silva-Sánchez J., Garza-Ramos U., Barrios-Camacho H., López-Jácome L.E., Colin-Castro C.A. (2021). Drug Resistance Phenotypes and Genotypes in Mexico in Representative Gram-Negative Species: Results from the Infivar Network. PLoS ONE.

[34] Garza-Ramos U., Davila G., Gonzalez V., Alpuche-Aranda C., López-Collada V.R., Alcantar-Curiel D., Newton O., Silva-Sanchez J. (2009). The BlaSHV-5 Gene Is Encoded in a Compound Transposon Duplicated in Tandem in Enterobacter Cloacae. Clin. Microbiol. Infect..

[35] Robicsek A., Strahilevitz J., Sahm D.F., Jacoby G.A., Hooper D.C. (2006). Qnr Prevalence in Ceftazidime-Resistant *Enterobacteriaceae* Isolates from the United States. Antimicrob. Agents Chemother..

[36] Yamane K., Wachino J., Suzuki S., Arakawa Y. (2008). Plasmid-Mediated QepA Gene among *Escherichia coli* Clinical Isolates from Japan. Antimicrob. Agents Chemother..

[37] CLSI (2023). M100: *Performance Standards for Antimicrobial Susceptibility Testing.

[38] Mansan-Almeida R., Pereira A.L., Giugliano L.G. (2013). Diffusely Adherent Escherichia Colistrains Isolated from Children and Adults Constitute Two Different Populations. BMC Microbiol..

[39] Rodriguez-Angeles M.G. (2002). Principales Características y Diagnóstico de Los Grupos Patógenos de *Escherichia coli*. Salud Publica Mex..

[40] Aranda K.R.S., Fabbricotti S.H., Fagundes-Neto U., Scaletsky I.C.A. (2007). Single Multiplex Assay to Identify Simultaneously Enteropathogenic, Enteroaggregative, Enterotoxigenic, Enteroinvasive and Shiga Toxin-Producing *Escherichia coli* Strains in Brazilian Children. FEMS Microbiol. Lett..

[41] Franke J., Franke S., Schmidt H., Schwarzkopf A., Wieler L.H., Baljer G., Beutin L., Karch H. (1994). Nucleotide Sequence Analysis of Enteropathogenic *Escherichia coli* (EPEC) Adherence Factor Probe and Development of PCR for Rapid Detection of EPEC Harboring Virulence Plasmids. J. Clin. Microbiol..

[42] Doumith M., Day M., Ciesielczuk H., Hope R., Underwood A., Reynolds R., Wain J., Livermore D.M., Woodford N. (2015). Rapid Identification of Major *Escherichia coli* Sequence Types Causing Urinary Tract and Bloodstream Infections. J. Clin. Microbiol..

[43] Versalovic J., Koeuth T., Lupski R. (1991). Distribution of Repetitive DNA Sequences in Eubacteria and Application to Finerpriting of Bacterial Enomes. Nucleic Acids Res..

[44] Letunic I., Bork P. (2021). Interactive Tree of Life (ITOL) v5: An Online Tool for Phylogenetic Tree Display and Annotation. Nucleic Acids Res..

[45] Wilde C., Mazel D., Hochhut B., Middendorf B., Le Roux F., Carniel E., Dobrindt U., Hacker J. (2008). Delineation of the Recombination Sites Necessary for Integration of Pathogenicity Islands II and III into the *Escherichia coli* 536 Chromosome. Mol. Microbiol..

[46] Navidinia M., Najar Peerayeh S., Fallah F., Bakhshi B., Adabian S., Alimehr S., Gholinejad Z. (2013). Distribution of the Pathogenicity Islands Markers (PAIs) in Uropathogenic *E.coli* Isolated from Children in Mofid Children Hospital. Arch. Pediatr. Infect. Dis..

[47] Chittò M., Berger M., Klotz L., Dobrindt U. (2020). Sub-Inhibitory Concentrations of SOS-Response Inducing Antibiotics Stimulate Integrase Expression and Excision of Pathogenicity Islands in Uropathogenic *Escherichia coli* Strain 536. Int. J. Med. Microbiol..

[48] Firoozeh F., Soleimani-Moorchekhorti L., Zibaei M. (2017). Evaluation of Pathogenicity Islands in Uropathogenic *Escherichia coli* Isolated from Patients with Urinary Catheters. J. Infect. Dev. Ctries..

[49] Dobrindt U., Blum-Oehler G., Nagy G., Schneider G., Johann A., Gottschalk G., Hacker J. (2002). Genetic Structure and Distribution of Four Pathogenicity Islands (PAI I 536 to PAI IV 536) of Uropathogenic *Escherichia coli* Strain 536. Infect. Immun..

[50] Schneider G., Dobrindt U., Brüggemann H., Nagy G., Janke B., Blum-Oehler G., Buchrieser C., Gottschalk G., Emödy L., Hacker J. (2004). The Pathogenicity Island-Associated K15 Capsule Determinant Exhibits a Novel Genetic Structure and Correlates with Virulence in Uropathogenic *Escherichia coli* Strain 536. Infect. Immun..

[51] Dahmen S., Métayer V., Gay E., Madec J.-Y., Haenni M. (2013). Characterization of Extended-Spectrum Beta-Lactamase (ESBL)-Carrying Plasmids and Clones of Enterobacteriaceae Causing Cattle Mastitis in France. Vet. Microbiol..

[52] Paniagua-Contreras G.L., Monroy-Pérez E., Bautista A., Reyes R., Vicente A., Vaca-Paniagua F., Díaz C.E., Martínez S., Domínguez P., García L.R. (2018). Multiple Antibiotic Resistances and Virulence Markers of Uropathogenic *Escherichia coli* from Mexico. Pathog. Glob. Health.

[53] Galindo-Méndez M. (2018). Caracterización Molecular y Patrón de Susceptibilidad Antimicrobiana de *Escherichia coli* Productora de β-Lactamasas de Espectro Extendido en Infección del Tracto Urinario Adquirida en La Comunidad. Rev. Chil. Infectología.

[54] Alcántar-Curiel M.D., Alpuche-Aranda C.M., Varona-Bobadilla H.J., Gayosso-Vázquez C., Jarillo-Quijada M.D., Frías-Mendivil M., Sanjuan-Padrón L., Santos-Preciado J.I. (2015). Risk Factors for Extended-Spectrumb-Lactamases-Producing Escherichia Coliurinary Tract Infections in a Tertiary Hospital. Salud Publica Mex..

[55] Gallegos-Miranda V., Garza-Ramos U., Bolado-Martínez E., Navarro-Navarro M., Félix-Murray K.R., Carmen M., Sanchez-Martinez G., Dúran-Bedolla J., Silva-Sánchez J., Garza-Ramos U. (2020). ESBL-Producing *Escherichia coli* and *Klebsiella pneumoniae* from Health-Care Institutions in Mexico. J. Chemother..

[56] Morfín-Otero R., Mendoza-Olazarán S., Silva-Sánchez J., Rodríguez-Noriega E., Laca-Díaz J., Tinoco-Carrillo P., Petersen L., López P., Reyna-Flores F., Alcantar-Curiel D. (2013). Characterization of *Enterobacteriaceae* Isolates Obtained from a Tertiary Care Hospital in Mexico, Which Produce Extended-Spectrum b-Lactamase. Microb. Drug Resist..

[57] Reyna-Flores F., Barrios H., Garza-Ramos U., Sánchez-Pérez A., Rojas-Moreno T., Uribe-Salas F.J., Fagundo-Sierra R., Silva-Sanchez J. (2013). Molecular Epidemiology of *Escherichia coli* O25b-ST131 Isolates Causing Community-Acquired UTIs in Mexico. Diagn. Microbiol. Infect. Dis..

[58] Paniagua-Contreras G.L., Monroy-Pérez E., Díaz-Velásquez C.E., Uribe-García A., Labastida A., Peñaloza-Figueroa F., Domínguez-Trejo P., García L.R., Vaca-Paniagua F., Vaca S. (2019). Whole-Genome Sequence Analysis of Multidrug-Resistant Uropathogenic Strains of *Escherichia coli* from Mexico. Infect. Drug Resist..

[59] Drinkovic D., Morris A.J., Dyet K., Bakker S., Heffernan H. (2015). Plasmid-Mediated AmpC Beta-Lactamase-Producing *Escherichia coli* Causing Urinary Tract Infection in the Auckland Community Likely to Be Resistant to Commonly Prescribed Antimicrobials. N. Z. Med. J..

[60] Ehsan B., Haque A., Qasim M., Ali A., Sarwar Y. (2023). High Prevalence of Extensively Drug Resistant and Extended Spectrum Beta Lactamases (ESBLs) Producing Uropathogenic *Escherichia coli* Isolated from Faisalabad, Pakistan. World J. Microbiol. Biotechnol..

[61] Ramírez-Castillo F.Y., Moreno-Flores A.C., Avelar-González F.J., Márquez-Díaz F., Harel J., Guerrero-Barrera A.L. (2018). An Evaluation of Multidrug-Resistant *Escherichia coli* Isolates in Urinary Tract Infections from Aguascalientes, Mexico: Cross-Sectional Study. Ann. Clin. Microbiol. Antimicrob..

[62] Robicsek A., Strahilevitz J., Jacoby G.A., Macielag M., Abbanat D., Hye Park C., Bush K., Hooper D.C. (2006). Fluoroquinolone-Modifying Enzyme: A New Adaptation of a Common Aminoglycoside Acetyltransferase. Nat. Med..

[63] Frasson I., Cavallaro A., Bergo C., Richter S.N., Palù G. (2011). Prevalence of Aac(6’)-Ib-Cr Plasmid-Mediated and Chromosome-Encoded Fluoroquinolone Resistance in *Enterobacteriaceae* in Italy. Gut Pathog..

[64] Seni J., Falgenhauer L., Simeo N., Mirambo M.M., Imirzalioglu C., Matee M., Rweyemamu M., Chakraborty T., Mshana S.E. (2016). Multiple ESBL-Producing *Escherichia coli* Sequence Types Carrying Quinolone and Aminoglycoside Resistance Genes Circulating in Companion and Domestic Farm Animals in Mwanza, Tanzania, Harbor Commonly Occurring Plasmids. Front. Microbiol..

[65] Juraschek K., Malekzadah J., Malorny B., Käsbohrer A., Schwarz S., Meemken D., Hammerl J.A. (2022). Characterization of QnrB-Carrying Plasmids from ESBL- and Non-ESBL-Producing *Escherichia coli*. BMC Genom..

[66] Riley L.W. (2014). Pandemic Lineages of Extraintestinal Pathogenic *Escherichia coli*. Clin. Microbiol. Infect..

[67] Moreira da Silva R.C.R., de Oliveira Martins Júnior P., Gonçalves L.F., de Paulo Martins V., de Melo A.B.F., Pitondo-Silva A., de Campos T.A. (2017). Ciprofloxacin Resistance in Uropathogenic *Escherichia coli* Isolates Causing Community-Acquired Urinary Infections in Brasília, Brazil. J. Glob. Antimicrob. Resist..

[68] Demirci M., Ünlü Ö., İstanbullu Tosun A. (2019). Detection of O25b-ST131 Clone, CTX-M-1 and CTX-M-15 Genes via Real-Time PCR in *Escherichia coli* Strains in Patients with UTIs Obtained from a University Hospital in Istanbul. J. Infect. Public Health.

[69] Hussain A., Ewers C., Nandanwar N., Guenther S., Jadhav S., Wieler L.H., Ahmed N. (2012). Multiresistant Uropathogenic *Escherichia coli* from a Region in India Where Urinary Tract Infections Are Endemic: Genotypic and Phenotypic Characteristics of Sequence Type 131 Isolates of the CTX-M-15 Extended-Spectrum-β-Lactamase-Producing Lineage. Antimicrob. Agents Chemother..

[70] Valiatti T.B., Santos F.F., Santos A.C.M., Nascimento J.A.S., Silva R.M., Carvalho E., Sinigaglia R., Gomes T.A.T. (2020). Genetic and Virulence Characteristics of a Hybrid Atypical Enteropathogenic and Uropathogenic *Escherichia coli* (AEPEC/UPEC) Strain. Front. Cell. Infect. Microbiol..

[71] Tanabe R.H.S., Dias R.C.B., Orsi H., de Lira D.R.P., Vieira M.A., dos Santos L.F., Ferreira A.M., Rall V.L.M., Mondelli A.L., Gomes T.A.T. (2022). Characterization of Uropathogenic *Escherichia coli* Reveals Hybrid Isolates of Uropathogenic and Diarrheagenic (UPEC/DEC) E. Coli. Microorganisms.

[72] Pérez Ama A. (2018). Estudio de Las Propiedades de Virulencia en Cepas de Escherichia coli Uropatógena.

[73] de M. Santos A.C., Santos F.F., Silva R.M., Gomes T.A.T. (2020). Diversity of Hybrid- and Hetero-Pathogenic *Escherichia coli* and Their Potential Implication in More Severe Diseases. Front. Cell. Infect. Microbiol..

