# Supplementary material for: Uropathogenic E. coli and Hybrid Pathotypes in Mexican Women with Urinary Tract Infections: A Comprehensive Molecular and Phenotypic Overview
Source: Curr Issues Mol Biol. 2024 Jun 13;46(6):5909–28. doi: 10.3390/cimb46060353 (PMC11202577; doi:10.3390/cimb46060353)
Supplement: Supplementary file 1 [file cimb-46-00353-s001.zip › cimb-3015948-supplementary.pdf]

Table S1. Oligonucleotide sequences and conditions used in this study.

Product	Secuence (5'-3')	Size (bp)	Tm°C	Ref
ERIC	ATGTAAGCTCCTGGGGGGATTCAC<br>AAGTAAGTGACTGGGGGGTGAGCG	Variable	50	(Versalovic et al., 1991)
ybbW	TGATTGGCAAAATCTGGCCG<br>ATACTGGCAATCAGTACGCC	670	60	(Carreón, 2019; Walker et al., 2017)
PAI III_{536}[a]	F: CGGGCATGCATCAATTATCTTTG<br>R: TGTGTAGATGCAGTCACTCCG	200	55	(Sabate et al., 2006)
PAI IV_{536}[a]	F: AAGGATTCGCTGTTACCGGAC<br>R: TCGTCGGGCAGCGTTTCTTCT	300		
PAI II_{CFT073}[a]	F: ATGGATGTTGTATCGCGC<br>R: ACGAGCATGTGGATCTGC	420		
PAI I_{536}[a]	F: TAATGCCGGAGATTCATTGTC<br>R: AGGATTTGTCTCAGGGCTTT	1,800		
PAI II_{536}[a]	F: CATGTCCAAAGCTCGAGCC<br>R: CTACGTCAGGCTGGCTTTG	1,000		
PAI I_{J96}[a]	F: TCGTGCTCAGGTCCGGAATTT<br>R: TGGCATCCCACATTATCG	400		
PAI II_{J96}[a]	F: GGATCCATGAAAACATGGTTAATGGG<br>R: GATATTTTTGTTGCCATTGGTTACC	2,300		
PAI I_{CFT073}[a]	F: GGACATCCTGTTACAGCGCGCA<br>R: TCGCCACCAATCACAGCGAAC	925		
*bla*CTX-M-1 y 8[b]	F: TGTGCAGYACCAGTAARGYKATG<br>R: TARRTSACCAGAAYVAGCGGC	583	55°C	(Arlet, 1991; Garza-González et al., 2021)
*bla*CTX-M-2[b]	F: CGAGTGGCAGTACCAGTAAGG<br>R: CGATATCGTTGGTGGTGC	540		
*bla*CTX-M-9[b]	F: ATGGTGACAAAGAGAGTGCAA<br>R: AATATCATTGGTGGTGCCGTAG	747		
*bla*CTX-M-15[b]	F: GCGGCCATGATAGGTACG<br>R: AAAGTAAGTCACAATAACCAGCG	786		
*bla*TEM[b]	F: CAACATTTTCGTGTCGCCC<br>R: GCTTAATCAGTGAGGCACC	844		
*bla*SHV[b]	F: TATTATCTCCCTGTTAGCCA<br>R: CGCTCTGCTTTGTTATTC	783	58°C	
qepA[b]	F: GCAGGTCCAGCAGCGGGTAG<br>R: CAACTGCTTGAGCCCGTAG	617	60°C	(Skočková et al., 2015; Wang et al., 2008)
qnrB[b]	F: GATCGTGAAAGCCAGAAAGG<br>R: ACGATGCCTGGTAGTTGTCC	482	53°C	
aac(6')-Ib-cr[b]	F: TTGCGATGCTCTATGAGTGGCTA<br>R: CTCGAATGCCTGGCGTGTTT	469	54°C	
daaE[c]	F: TGACTGTGACCGAAGAGTGC<br>R: TTAGTTCGTCCAGTAACCCCC	380	48°C	(Mansan-Almeida et al., 2013)
pEAE[c]	F: CAGGGTAAAAGAAAGATGATAA<br>R: TATGGGGACCATGTATTATCA	1,087	58°C	(Alikhani et al., 2006)
bfpA[c]	F: AATGGTGCTTGCGCTTGCTGC<br>R: GCCGCTTTATCCAACCTGGTA	326	60°C	(Beutin et al., 2003)
EAEC[c]	F: CTGGCGAAAGACTGTATCAT<br>R: CAATGTATAGAAATCGCTGTT	630		(Schmidt et al., 1995)
ST73[d]	F: TGGTTTTACCATTTTGTCGGA<br>R: GGAAATCGTTGATGTTGGCT	490pb	60°C	(Doumith et al., 2015)
ST131[d]	F: GACTGCATTTCGTCGCCATA<br>R: CCGGCGGCATCATAATGAAA	310pb		
ST95[d]	F: ACTAATCAGGATGGCGAGAC<br>R: ATCACGCCCATTAATCCAGT	200pb		
ST69[d]	F: ATCTGGAGGCAACAAGCATA<br>R: AGAGAAAGGGCGTTCAGAAT	104pb		

Implemented primers for: [a]: Pathogenicity islands; [b]: Antibiotic Resistance; [c]: Pathotypes; [d]: Sequence types.

Table S2. Previously Reported Resistance Profiles of Analyzed Clinical Isolates

ID	Resistant Profile
8, 9	-
37	AMC, AMS
21	AMK, CFZ, CTX, CRO, AMC, AMS
14	AMK, CIP, CTX, CRO
19	AMK, CTX, AMC
4	AMK, GM
7	AMK, GM, AMC, MEM
22	AMK, GM, CFZ, CTX
28	AMK, GM, CFZ, CTX, CRO, AMC, AMS
39	AMK, GM, CIP, NOR, CFZ, CTX, CRO, AMC, AMS, IMP
27	AMK, GM, CIP, NOR, LVX, CX, CFZ, CTX, CRO, ATM, AMC, MEM
32, 34	AMK, GM, CIP, NOR, LVX, CFZ, CTX, CRO, AMC, AMS
29	AMK, GM, CIP, NOR, LVX, CFZ, CTX, CRO, ATM, AMC
1	AMK, GM, CIP, NOR, LVX, CTX, AMC, AMS
35	AMK, GM, CIP, NOR, LVX, CX, CFZ, CTX, CRO, AMC, IMP
36	AMK, GM, CIP, NOR, LVX, CX, CFZ, CTX, CRO, ATM, AMC
17	AMK, GM, CTX
12, 26	AMK, GM, CFZ, AMC, AMS
23, 25	AMK, GM, CTX, CRO, AMC
3	AMK, GM, CTX, CRO, AMC, AMS
20	AMK, GM, CTX, CRO, ATM, AMC
33	AMK, GM, CTX, CRO, ATM, AMC, AMS
24	AMK, GM, CX, CFZ, CTX, CRO, AMC
30	CFZ, CTX, AMC, AMS
40	CIP, NOR, LVX, CFZ, CTX, CRO, FEP, ATM, AMC, AMS, IMP
18	CTX, AMC, AMS
13	CTX, ATM
5	CX, CFZ, CTX, CRO, FEP, ATM, AMC, AMS
10	GM, CFZ, CTX
16	GM, CFX, CTX, AMC
6	GM, CIP, NOR, LVX, AMC
11	GM, CIP, NOR, LVX, CFZ, CTX, AMC
2	GM, CIP, NOR, LVX, CFZ, CTX, CRO, FEP, ATM, AMC, AMS
38	GM, LVX, TSX, AMP, CFX, FEP, ATM, AMC, AMS
31	GM, CIP, NOR, LVX, CX, CFZ, CTX, CRO, AMC
15	GM, CTX, AMC

AMK: Amikacin; **GM:** Gentamicin; **CIP:** Ciprofloxacin; **NOR:** Norfloxacin; **LVX:** Levofloxacin; **CX:** Cefoxitin; **CFZ:** Ceftazidime; **CTX:** Cefotaxime; **CRO:** Ceftriaxone; **FEP:** Cefepime; **ATM:** Aztreonam; **AMC:** Amoxicillin-Clavulánic Acid; **AMS:** Ampicillin-Sulbactam; **MEM:** Meropenem; **IMP:** Imipenem; **ETP:** Ertapenem.

Table S3. Correlation between antibiotic resistance phenotypes and genotypes.

Variable		AMK	GM	CIP	NOR	LVX	*qepA*	*qnrB*	*aac(6')-Ib*	ESBL$_{CTX}$	ESBL$_{CFZ}$	ESBL$_{CRO}$	ESBL$_{FEP}$	ESBL$_{ATM}$
1. AMK	*r*	—												
	p	—												
2. GM	*r*	0.9916	—											
	p	< .001	—											
3. CIP	*r*	0.9691	0.9757	—										
	P	< .001	< .001	—										
4. NOR	*r*	0.9652	0.9753	0.9972	—									
	p	< .001	< .001	< .001	—									
5. LVX	*r*	0.9618	0.9753	0.9916	0.994	—								
	p	< .001	< .001	< .001	< .001	—								
6. *qepA*	*r*	0.9491	0.9632	0.9784	0.9807	0.9884	—							
	p	< .001	< .001	< .001	< .001	< .001	—							
7. *qnrB*	*r*	0.9557	0.9575	0.9802	0.9744	0.9744	0.9614	—						
	p	< .001	< .001	< .001	< .001	< .001	< .001	—						
8. *aac(6')-Ib*	*r*	0.9691	0.9757	1	0.9972	0.9916	0.9784	0.9802	—					
	p	< .001	< .001	< .001	< .001	< .001	< .001	< .001	—					
9. ESBL$_{CTX}$	*r*	0.9758	0.9809	0.9669	0.9624	0.9582	0.9438	0.9469	0.9669	—				
	p	< .001	< .001	< .001	< .001	< .001	< .001	< .001	< .001	—				
10. ESBL$_{CFZ}$	*r*	0.948	0.9461	0.9379	0.938	0.938	0.9139	0.9252	0.9379	0.9575	—			
	p	< .001	< .001	< .001	< .001	< .001	< .001	< .001	< .001	< .001	—			
11. ESBL$_{CRO}$	*r*	0.9604	0.9575	0.9483	0.9487	0.9401	0.9173	0.9269	0.9483	0.9588	0.966	—		
	p	< .001	< .001	< .001	< .001	< .001	< .001	< .001	< .001	< .001	< .001	—		
12. ESBL$_{FEP}$	*r*	0.8489	0.871	0.8681	0.8694	0.89	0.8404	0.8391	0.8681	0.864	0.8746	0.8779	—	
	p	< .001	< .001	< .001	< .001	< .001	< .001	< .001	< .001	< .001	< .001	< .001	—	
13. ESBL$_{ATM}$	*r*	0.919	0.9304	0.9194	0.9195	0.9321	0.9039	0.8847	0.9194	0.9365	0.9008	0.9203	0.93	—
	p	< .001	< .001	< .001	< .001	< .001	< .001	< .001	< .001	< .001	< .001	< .001	< .001	—

[R]**:** Resistance phenotype; **AMK:** Amikacin; **GM:** Gentamicin; **CIP:** Ciprofloxacin; **NOR:** Norfloxacin; **LVX:** Levofloxacin; ***qepA*:** Quinolone efflux pump associated gene; ***qnrB*:** Quinolone resistant associated gene; ***aac(6')-Ib:*** Acetylase associated gene; **ESBL:** ESBL positive phenotype; **CTX:** Cefotaxime; **CFZ:** Ceftazidime; **CRO:** Ceftriaxone; **FEP:** Cefepime; **ATM:** Aztreonam. The *r* and *p* values were obtained by the Pearson correlation test.

Table S4. Statistical Analysis for Association Between Plasmids Presence and Virulence or Resistance Evaluated Features

Feature	Isolates with Plasmids %(n= 23)	Isolates without Plasmids %(n= 17)	*p* value
Pathogenicity Islands			
PAI III_{536}	17.4 (4)	41.2 (7)	0.15
PAI IV_{536}	52.2 (12)	53 (9)	1
PAI II_{CFT073}	22 (5)	53 (9)	0.06
PAI II_{536}	17.4 (4)	6 (1)	0.4
PAI I_{J96}	35 (8)	35.3 (6)	1
PAI I_{CFT073}	26 (6)	29.4 (5)	1
PAI I_{536}	(0)	12 (2)	0.2
PAI II_{J96}	9 (2)	12 (2)	1
ESBL Production Phenotypes			
$ESBL_{CTX}$	48 (11)	35 (8)	1
$ESBL_{CFZ}$	17.4 (4)	24 (4)	0.7
$ESBL_{CRO}$	17.4 (4)	29.4 (5)	0.45
ESBLFEP	9 (2)	6 (1)	1
$ESBL_{ATM}$	13 (3)	18 (3)	1
Carbapenemase Production Phenotypes			
CAR_{MEM}	9 (2)	6 (1)	1
CAR_{IMP}	9 (2)	(0)	0.5
ESBL Associated Genes			
$bla_{CTX\text{-}M\ 1\ y\ 8}$	48 (11)	41.2 (7)	0.75
$bla_{CTX\text{-}M\text{-}9}$	17.4 (4)	12 (2)	1
bla_{TEM}	39.1(9)	41.2 (7)	1
$bla_{CTX\text{-}M\text{-}2}$	35 (8)	53 (9)	0.33
$bla_{CTX\text{-}M\text{-}15}$	4.3 (1)	12 (2)	0.6
Fluoroquinolones and Aminoglycosides Resistance Genes			
qepA	30.4 (7)	18 (3)	0.5
aac(6')-Ib	45 (10)	24 (4)	0.31
qnrB	30.4 (7)	12 (2)	0.26
Sequence Types			
ST73	4.3 (1)	(0)	1
ST131	70 (16)	35 (8)	0.2
ST69	9 (2)	18 (3)	0.6
Antibiotic Resistance Phenotypes			
AMK	61 (14)	59 (10)	1
GM	65.2 (15)	82.3 (14)	0.3
CIP	45 (10)	24 (4)	0.31
NOR	39.1 (9)	24 (4)	0.33
LVX	39.1 (9)	24 (4)	0.33
CX	17.4 (4)	12 (2)	1
CFZ	48 (11)	41.2 (7)	0.75
CTX	83 (19)	82.3 (14)	1
CRO	45 (10)	59 (10)	0.52
FEP	9 (2)	12 (2)	1
ATM	26 (6)	24 (4)	1
AMC	74 (17)	88.2 (15)	0.42
AMS	45 (10)	41.2 (7)	1
MEM	4.3 (1)	6 (1)	1
IMP	13 (3)	(0)	0.25

p values were obtained by Fisher Exact Test.

Table S5. Statistical Analysis for Correlation Between Plasmid Sizes and Resistance or Virulence Features

Size (kb)	Feature	*r* value	*p* value
1-5 kb	CIP	0.3355	0.0343
	AMC	-0.3851	0.0141
	aac(6')-Ib	0.3355	0.0343
	qnrB	0.3236	0.0417
6-10 kb	CIP	0.368	0.0195
	aac(6')-Ib	0.368	0.0195
	qnrB	0.357	0.0237
21-30 kb	CTX	-0.4981	0.0011
	AMC	-0.4588	0.0029
51-100 kb	FEP	0.378	0.0162
101-120 kb	LVX	0.3306	0.0372
	FEP	0.6882	< .001
	ATM	0.3974	0.0111
	IMP	0.3702	0.0187
	$ESBL_{FEP}$	0.3691	0.0207
	$ESBL_{ATM}$	0.5461	< .001
	qepA	0.3974	0.0111

The *r* and *p* values were obtained by Pearson Correlation Test. Only significant results are shown.

Table S6. Virulence genes, PAI profiles, DEC genes and adherence patterns of UPEC clinical isolates.

ID	Virulence Genes/PAI Profiles	DEC Genes	Adherence Profile
1	*fimH, papG-II, sat, hlyA, traT, iroN, fyuA,I ha,feoB,*<br>PAI II_{536}, PAI III_{536}, PAI IV_{536}, PAI I_{CFT073}, PAI II_{CFT073}	pCVD432, *eaeA, daaE, bfpA*	Lo/Bs
2	*fimH,fliCD,hlyA,iucD,iutA,feoB,*<br>PAI II_{536}, PAI III_{536}, PAI IV_{536}, PAI I_{CFT073}, PAI II_{CFT073}	pCVD432, *bfpA*	Bs/Di
3	*fimH, papG-II, fliCD,sat,vat,traT,agn43,iroN,iucD,fyuA,iha,feoB*	pCVD432, *bfpA*	Bs/Lo/Ag
4	*fimH, papG-II, fliCD, sat, vat,traT,agn43,iroN,fyuA,iha,feoB,*<br>PAI III_{536}, PAI IV_{536}, PAI I_{J96}	*pCVD432, bfpA*	Bs/Lo
5	*fimH,fliCD,sat,kpsM,traT,iucD,feoB,*<br>PAI III_{536}, PAI IV_{536}, PAI II_{CFT073}	*daaE, bfpA*	Ag
6	*fimH, fliCD, sat, vat,traT,agn43,iucD,fyuA,iha,feoB,*<br>PAI III_{536}, PAI IV_{536}, PAI II_{CFT073}	*daaE, bfpA*	Lo/Ag/Bs
7	*fimH,agn43,iutA*	pCVD432, *bfpA*	Bs
8	*fimH,fliCD,hlyA,kpsM,traT,feoB,*<br>PAI III_{536}, PAI II_{J96}, PAI I_{CFT073}	-	Bs/Lo
9	*papC,fimH,sfaD/focC,papG-II, fliCD,kpsM,vat,cnf1,iroN,iucD,fyuA,feoB*<br>PAI I_{J96}, PAIII_{J96}, PAI I_{CFT073}	*daaE, bfpA*	Bs/Ag
10	*fimH,papG-II,fliCD,vat,traT,iroN,fyuAfeoB,*<br>PAI IV_{536}, PAI I_{J96}	*bfpA*	Bs/Lo
11	*fimH,sat,hlyA,kpsM,cnf-1,agn43,iucD,feoB,*<br>PAI IV_{536}	*daaE, bfpA*	Bs
12	*papC,fimH,papG-II,sat,cnf-1, traT,agn43,iroN,iucD,fyuA,iha,feoB,*<br>PAI III_{536}, PAI IV_{536}	-	Lo
13	*fimH, papG-II, fliCD, traT, feoB*	*bfpA*	Lo/Ag
14	*fimH,kpsM,iucD,feoB,*<br>PAI II_{536}	*bfpA*	Bs
15	*fimH, fliCD, cnf-1, traT,agn43,iucD,iha,feoB,*<br>PAI II_{536}	-	Bs
16	*fimH,fliCD,hlyA,iucD,iha,iutA,feoB*	*bfpA*	Bs/Lo
17	*fimH,papG-II,fliCD,hlyA,iucD,fyuA,iha,feoB*	*bfpA*	Bs
18	*fimH,papG-II,fliCD,iroN,fyuA,iutA,feoB*	*bfpA*	Bs
19	*fimH, fliCD, sat, iroN, iucD, fyuA, feoB,*<br>PAI I_{J96}, PAI I_{CFT073}	-	Bs/Lo/Ag
20	*fimH,papG-II,fliCD,hlyA,traT,feoB,*<br>PAI III_{536}, PAI II_{CFT073}	*bfpA*	Bs/Lo/Di/ Ag

ID: Isolate; **PAI:** Pathogenicity islands; **Bs:** Bricks pattern; **Lo:** Localized pattern; **Di:** Diffuse pattern; **Ag:** Aggregative pattern. ***fimH*:** Type 1 pilus Adhesin; ***sfaD/focC*:** S and Dra fimbriae; ***papC*:** Type P pilus chaperone; ***papG-II*:** Type P pilus Adhesin allele 2; ***fliCD*:** Flagellin subunit/flagellar cap; ***hlyA*:** α-hemolysin; ***kpsM*:** Capsular variant; ***sat*:** Autotransporter secreted toxin; ***agn43*:** Antigen 43; vat: Vacuolating autotransporter toxin; ***cnf-1*:** Necrotizing cytotoxic factor; ***traT*:** Complement resistance associated protein; ***fyuA*:** Ferric yersiniabactin uptake receptor; ***iucD*:** Aerobactin; ***iroN*:** Salmocheline receptor; ***iutA*:** Aerobactin receptor; ***feoB*:** Ferrous iron transporter; ***iha*:** IrgA homologue Adhesin/enterobactin receptor.

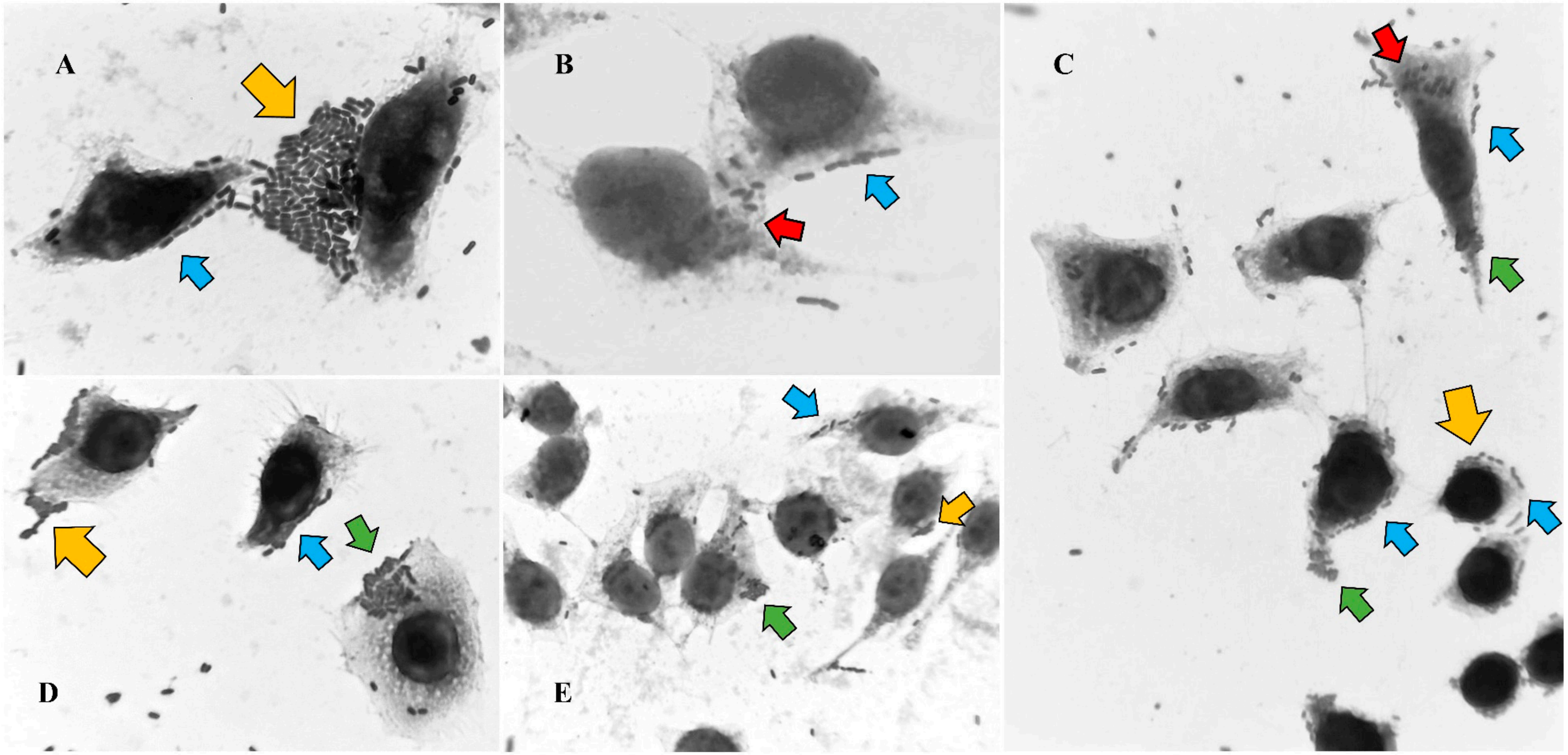

Figure S1. Mixed adherence pattern in clinical isolates of *E. coli*. **A)** Ec-9, shown a mixed aggregative and stacked-brick patterns; **B)** Ec-2, shown a mixed diffuse adherence and stacked-brick patterns; **C)** Ec-16, shown a mixed aggregative, diffuse adherence, and stacked-brick patterns; D) Ec-16, shown an aggregative, localized, and steacked-brick patterns; E) Ec-19, shown an aggregative, localized, and sticked-brick patterns. Yellow arrow indicated an aggregative pattern; Blue arrows are for steacked-breack pattern; Green arrows shown the localized pattern; Red arrows, indicated the diffuse adherence pattern.
